# Targeting the N-acetyltransferase 10/DKK2 axis enhances CD8^+^ T cell antitumor activity in colorectal cancer models

**DOI:** 10.1172/JCI196722

**Published:** 2026-01-16

**Authors:** Mengmeng Li, Xiaoya Zhao, Jun Wu, Shimeng Zhou, Yao Fu, Chen Chen, Zhuang Ma, Jiawen Xu, Yun Qian, Zhangding Wang, Bo Wang, Qiang Wang, Qingqing Ding, Changyu Chen, Honggang Wang, Xiaozhong Yang, Weijie Dai, Wenjie Zhang, Shouyu Wang

**Affiliations:** 1 Department of Hepatobiliary Surgery, The First Affiliated Hospital of Anhui Medical University, MOE Innovation Center for Basic Research in Tumor Immunotherapy, Anhui Province Key Laboratory of Tumor Immune Microenvironment and Immunotherapy, Hefei, Anhui Province, China.; 2 Medical School of Nanjing University, Nanjing, Jiangsu Province, China.; 3 Department of Thoracic Surgery, Northern Jiangsu People’s Hospital, Yangzhou, China.; 4 Department of Pathology, The First Affiliated Hospital of Anhui Medical University, Hefei, Anhui Province, China.; 5 Department of Geriatric Oncology, The First Affiliated Hospital of Nanjing Medical University, Nanjing, Jiangsu Province, China.; 6 Department of General Surgery, The First Affiliated Hospital of Anhui Medical University, Hefei, Anhui Province, China.; 7 Department of Gastroenterology, The Affiliated Huaian No. 1 People’s Hospital of Nanjing Medical University, Huai’an, Jiangsu Province, China.

**Keywords:** Gastroenterology, Immunology, Oncology, Cancer immunotherapy, Colorectal cancer, Epigenetics

## Abstract

Despite overexpression of N-acetyltransferase 10 (NAT10) in colorectal cancer (CRC), its immunomodulatory role in the tumor microenvironment remains elusive. Here, we reveal that NAT10 promotes immune evasion through N4-acetylcytosine–dependent (ac4C-dependent) mRNA stabilization. Using syngeneic mouse models (MC38/CT-26), intestinal epithelial-cell specific *Nat10* conditional KO (*Nat10*^cKO^) mice, patient-derived organoids, and clinical specimens, we show that *Nat10* ablation enhanced CD8^+^ T cell–mediated antitumor immunity. Single-cell RNA-seq revealed increased cytotoxic CD8^+^ T cell infiltration in *Nat10*^cKO^ tumors, which was corroborated by the inverse correlation of tumoral NAT10 expression and CD8^+^ T cell number in clinical specimens. Multi-omics integration analysis identified DKK2 as the predominant NAT10-regulated transcript. NAT10 stabilized *DKK2* mRNA via ac4C modification, leading to high expression of the DKK2 protein. Secreted DKK2 engaged LRP6 receptors to activate AKT-mTOR signaling, inducing cholesterol accumulation in CD8^+^ T cells and impairing their cytotoxicity. Pharmacological NAT10 inhibition (Remodelin treatment) or DKK2 neutralization restored CD8^+^ T cell function and synergized with anti–PD-1 therapy. Our findings establish the NAT10/DKK2/LRP6/AKT-mTOR/cholesterol axis as a critical regulator of CD8^+^ T cell dysfunction in CRC, positioning NAT10/DKK2 as a potential target to enhance immunotherapy efficacy.

## Abstract 2

Targeting the NAT10/DKK2 axis suppresses colorectal cancer by enhancing CD8^+^ T cells antitumor activity

## Graphical abstract

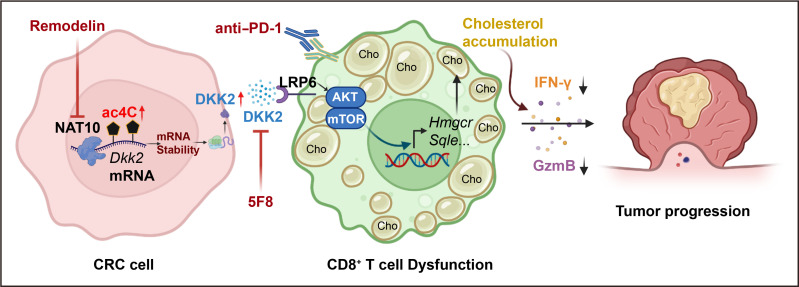

## Introduction

Colorectal cancer (CRC) remains a global health challenge, ranking as the third most prevalent malignancy and the second leading cause of cancer-related mortality worldwide. Despite advances in early detection, more than 20% of patients are diagnosed at an advanced stage, and these patients have a 5-year survival rate of 14% (1, 2). While immune checkpoint inhibitors have revolutionized cancer treatment in melanoma and lung cancer (3), their efficacy in treating CRC is largely restricted to microsatellite instability–high or mismatch repair deficiency subtypes, leaving most patients with microsatellite-stable tumors refractory to immunotherapy (3, 4). This stark therapeutic disparity underscores the critical need to elucidate the mechanisms of immune evasion and identify actionable targets to reprogram the tumor microenvironment (TME) in CRC.

Immunological imbalance within the TME is one of the critical hallmarks of cancer (5). The immunosuppressive TME in CRC is characterized by dysfunctional CD8^+^ T cells, which are the key mediators of antitumor immunity (6, 7). Initially, CD8^+^ T cells infiltrate tumors and specifically recognize tumor antigens to initiate cytotoxicity. However, tumor cells can evade immune surveillance by creating various immunosuppressive microenvironments, such as by downregulating the expression of MHC-I molecules, inhibiting the production of chemokines, or increasing the expression of inhibitory molecules such as PD-L1, all of which limit the infiltration, activation, and cytotoxicity of CD8^+^ T cells (8–10). Additionally, tumor cells can manipulate T cell metabolism to hinder antitumor immune responses. Metabolites within the TME, such as lactate, cholesterol, and fumarate, have been reported to inhibit antitumor immunity (11–14). However, the molecular drivers linking tumor-intrinsic pathways to cholesterol metabolism in T cell remain elusive.

Recently, RNA modifications have emerged as pivotal regulators of tumorigenesis and cancer progression. Various modifications, such as 6-methyladenine (m6A), N4-acetylcytosine (ac4C), 5-methylcytosine (m5C), 7-methylguanine (m7G), and 1-methyladenine (m1A), have been detected on mRNAs and noncoding RNAs. These dynamic and reversible chemical modifications have diverse biological functions, with their abundance and regulatory mechanisms varying across different diseases, including various cancer types (15). Among these, ac4C modification occurs mainly in the coding region (CDS) and 5′-noncoding region (5′-UTR) of RNAs, where it enhances RNA stability and translation efficiency (15). N-acetyltransferase 10 (NAT10) is the only known acetyltransferase that catalyzes ac4C modification. While NAT10 has been found to promote CRC growth via Wnt/β-catenin activation (16) and ferroptosis suppression (17), its immunomodulatory functions remain unexplored. Critically, few studies have addressed whether RNA acetylation governs tumor-immune crosstalk, representing a fundamental gap in the understanding of the epigenetic regulation of the TME in CRC.

Here, we identified NAT10 as a master regulator of CD8^+^ T cell dysfunction in CRC. Through integrated multi-omics and functional studies, we demonstrated that NAT10-mediated ac4C modification stabilized *DKK2* mRNA, driving its hypersecretion into the TME. Secreted DKK2 engaged LRP6 on CD8^+^ T cells to activate AKT-mTOR signaling, which induced pathological cholesterol accumulation and impaired cytotoxic effector functions. Importantly, pharmacological targeting of NAT10 or DKK2 synergizes with anti–PD-1 therapy, providing a potential combinatorial strategy to overcome immunotherapy resistance in CRC.

## Results

### NAT10 depletion attenuates tumor progression and activates antitumor immunity in CRC allografts.

**Figure 1:**
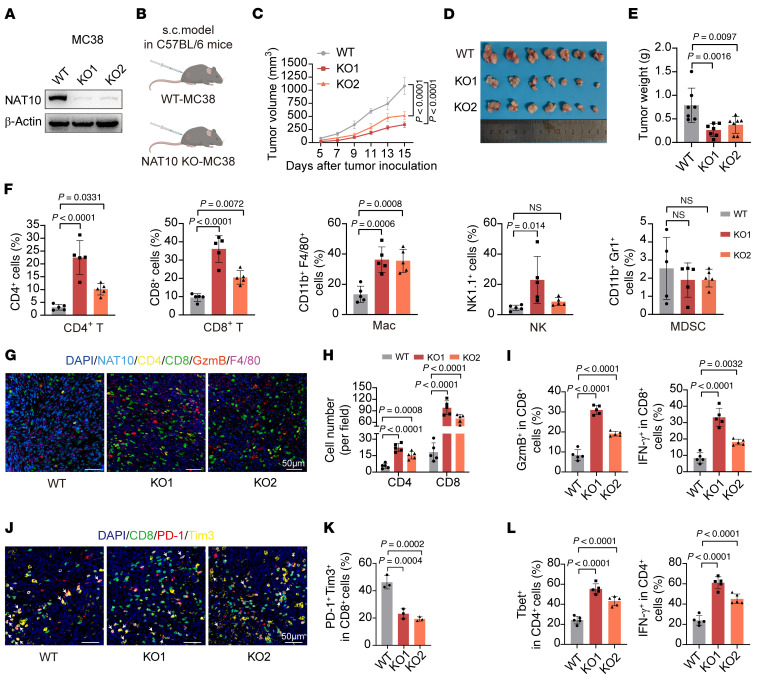
*NAT10* deficiency attenuates CRC progression and activates antitumor immunity in syngeneic allografts. (**A**) Western blot confirming *Nat10* KO in MC38 cells. (**B**) Schematic for the subcutaneous implantation of WT or *Nat10*-KO MC38 cells into C57BL/6 mice. (**C**–**E**), Tumor growth curves (mean ± SEM) (**C**), representative images of tumors from each group (**D**), and tumor weights (**E**) (*n* = 7 mice/group). (**F**) The composition of immune cells in tumors from the MC38 WT and *Nat10*-KO groups was determined via flow cytometry (*n* = 5 mice/group). The data are presented as the mean ± SD of indicated mice per group and are representative of 2 independent experiments (**C**–**F**). (**G**) Representative mIHC staining of Nat10, GzmB, CD4^+^ T cells, CD8^+^ T cells and macrophages in tumor sections (*n* = 5 mice/group). Scale bar: 50 μm. (**H**) Quantification of CD4^+^ T cell and CD8^+^ T cell densities in tumor sections (*n* = 5 mice/group). (**I**) Flow cytometric analysis of GzmB^+^ and IFN-γ^+^CD8^+^ T cell infiltration in tumors from the MC38 *Nat10*-WT and KO groups (*n* = 5 mice/group). (**J** and **K**) mIHC-based quantification of exhausted PD-1^+^ Tim-3^+^ CD8^+^ T cells. Representative images are shown in **J** (scale bar: 50 μm), and quantification analysis is shown in **K** (*n* = 3 mice/group). (**L**) Flow cytometry assessment of Tebt^+^ and IFN-γ^+^CD4^+^ T cell populations. Data are shown as the mean ± SD of indicated mice per group (**H**, **I**, **K**, and **L**). Statistical analysis was performed by 1-way ANOVA (**E**, **F**, **H**, **I**, **K**, and **L**) and 2-way ANOVA (**C**). ns, *P* ≥ 0.05. *P* < 0.05 was considered to indicate statistical significance.

To explore the role of Nat10 in modulating tumor immunity, we constructed stable *Nat10*-KO cell lines in murine CRC cells (MC38 and CT-26) using CRISPR/Cas9 (Figure 1A and Supplemental Figure 1F; supplemental material available online with this article; https://doi.org/10.1172/JCI196722DS1). Compared with the implantation of WT control cells, the subcutaneous implantation of MC38 *Nat10*-KO cells into immunodeficient and syngeneic immunocompetent mice suppressed tumor growth (Figure 1, B and C, and Supplemental Figure 1, A and B). However, the inhibitory effect of *Nat10* ablation on MC38 tumor growth was markedly greater in immunocompetent C57BL/6 mice than in immunodeficient BALB/c nude mice (Figure 1C and Supplemental Figure 1B), as reflected by reduced tumor volume and weight (Figure 1, D and E, and Supplemental Figure 1C). Similar suppression was observed in CT-26-derived allografts with *Nat10* depletion in BALB/c mice (Supplemental Figure 1, G–I), suggesting that the immunomodulatory functions of Nat10 depend on host immune competence.

Multicolor flow cytometry analysis of tumor-infiltrating immune cells revealed that *Nat10* deletion markedly increased CD8^+^ and CD4^+^ T cell infiltration and modestly elevated the numbers of NK cells and macrophages, whereas the myeloid-derived suppressor cell populations were unaltered (Figure 1F). Multiplex immunohistochemical staining confirmed increased CD8^+^ and CD4^+^ T cell proportions in *Nat10*-KO MC38 tumors (Figure 1, G and H). Functional characterization revealed increased numbers of granzyme B^+^ (GzmB), IFN-γ, and perforin^+^ CD8^+^ T cells (Figure 1I and Supplemental Figure 1D) and reduced numbers of PD-1^+^ im3^+^ and LAG3^+^ exhausted CD8^+^ T cells in *Nat10*-KO tumors (Figure 1, J and K, and Supplemental Figure 1E). Additionally, IFN-γ^+^ and Tbet^+^CD4^+^ T cell populations were amplified (Figure 1L). Consistent results were obtained in CT-26 allografts, where *Nat10* KO increased the accumulation and activity of CD8^+^ and CD4^+^ T cells, which were correlated with tumor growth suppression (Supplemental Figure 1, G–M). Collectively, *Nat10* deficiency reshaped the tumor immune microenvironment by promoting infiltration of effector T cells, enhancing their cytotoxic function, and mitigating their exhaustion, ultimately bolstering antitumor immunity in CRC models.

### Intestinal epithelial cell-specific Nat10 deficiency suppresses colorectal tumorigenesis and enhances CD8^+^ T cell–mediated antitumor immunity.

**Figure 2:**
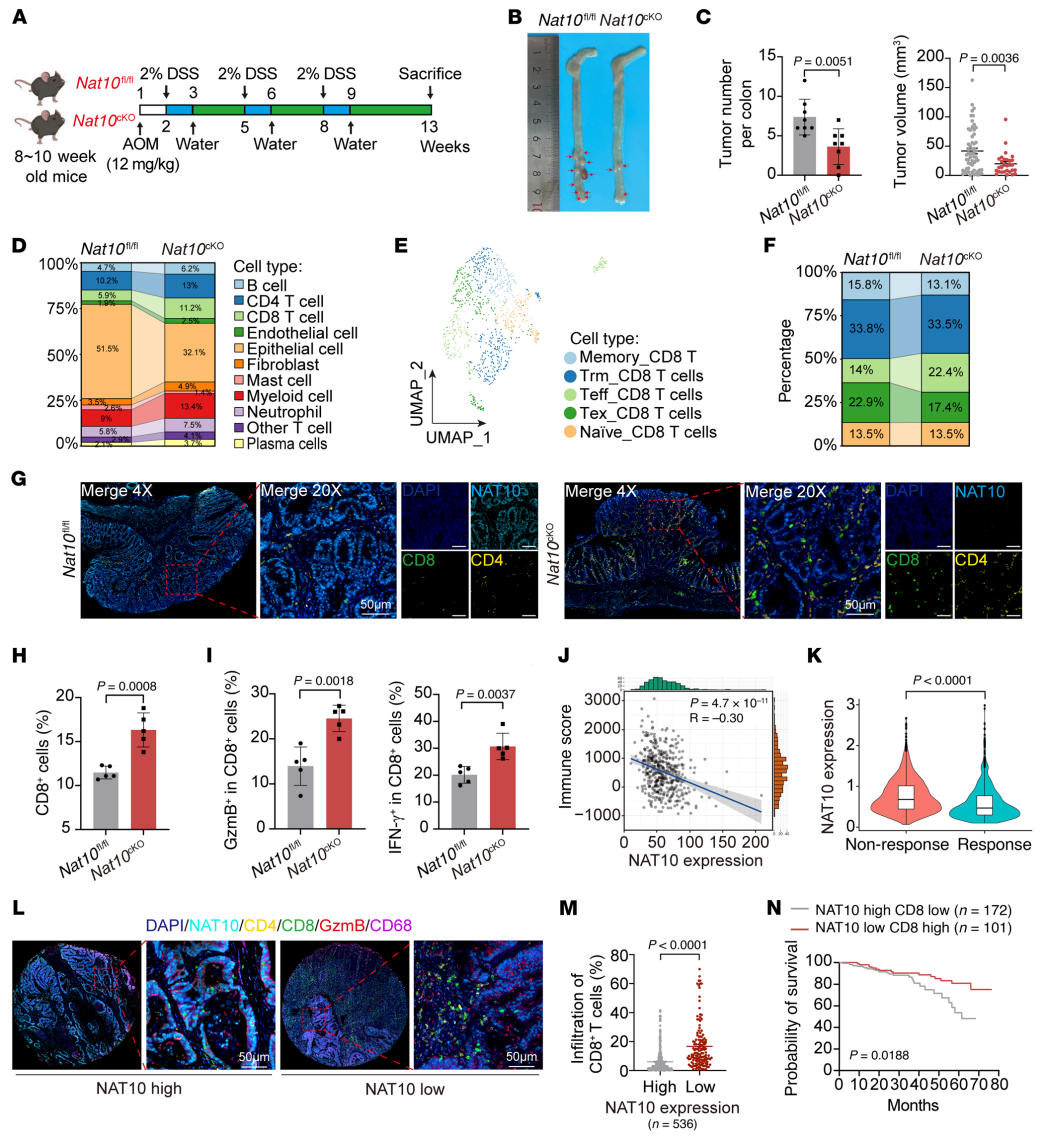
Intestinal epithelial cell-specific *Nat10* deficiency attenuates colorectal tumorigenesis and enhances CD8^+^ T cell–mediated antitumor immunity. (**A**) Schematic illustration of AOM/DSS-induced CRC in *Nat10*^fl/fl^ and *Nat10*^cKO^ mice. (**B** and **C**) Representative colon tumor (**B**) and tumor number and tumor volume (**C**) in *Nat10*^fl/fl^ and *Nat10*^cKO^ mice (*n* = 8 mice/group). (**D**) scRNA-seq analysis of the cell types from colon tumors in *Nat10*^fl/fl^ and *Nat10*^cKO^ mice. (**E** and **F**) UMAP plot of tumor-infiltrating CD8^+^ T cells subset and proportions of subset. Tex, exhausted T; Teff, effector T; Trm, tissue-resident memory T. (**G**) Representative mIHC staining of CD4^+^ T cells, CD8^+^ T cells, and macrophages in tumors from *Nat10*^fl/fl^ and *Nat10*^cKO^ mice (*n* = 5 mice/group). Scale bar: 50 μm. (**H** and **I**) Flow cytometric analysis of total CD8^+^ T cells (**H**) and GzmB^+^ or IFN-γ^+^ cytotoxic subsets (**I**) (*n* = 5 mice/group). (**J**) ESTIMATE algorithm analysis of the correlation between *Nat10* expression and the immune score in a TCGA CRC cohort (https://tcga-data.nci.nih.gov/tcga/) (*n* = 471). (**K**) Differential *NAT10* expression in tumors from immunotherapy-responsive versus nonresponsive patients with CRC (GSE205506 dataset). (**L**) Representative mIHC staining of NAT10, GzmB, CD4^+^ T cells, CD8^+^ T cells, and macrophages in human CRC tissue microarrays (*n* = 536). Scale bar: 50 μm. (**M**) Proportions of infiltrated CD8^+^ T cells in human CRC tumors with high or low NAT10 expression (*n* = 536) (mean ± SEM). (**N**) Kaplan-Meier survival curves stratified by NAT10 and CD8 coexpression. Data are shown as the mean ± SD of indicated mice per group (**C**, **H**, and **I**). Statistical analysis was performed by 2-tailed Student’s *t* test (**C**, **H**, **I**, and **M**) and log-rank test (**N**). ns, *P* ≥ 0.05. *P* < 0.05 was considered to indicate statistical significance.

To investigate the role of NAT10 in spontaneous colorectal carcinogenesis, we generated intestinal epithelium-specific *Nat10* conditional KO (*Nat10*^cKO^) mice (Supplemental Figure 2, A and B) and established an azoxymethane/dextran sodium sulfate-induced (AOM/DSS-induced) CRC model (Figure 2A). Compared with their WT *Nat10*^fl/fl^ littermates, *Nat10*^cKO^ mice presented a reduced intestinal tumor burden and smaller tumor volumes (Figure 2, B and C). Additionally, immunohistochemical staining for Ki67 revealed a reduced proportion of proliferating tumor cells in *Nat10*^cKO^ tumors (Supplemental Figure 2C), further supporting the tumor-suppressive effect of intestinal *Nat10* ablation.

To further investigate the effect of Nat10 on the CRC TME, we isolated colorectal tumor tissues from *Nat10*^fl/fl^ and *Nat10*^cKO^ mice and performed single-cell RNA-seq (scRNA-seq). Compared with that in the *Nat10*^fl/fl^ group, CD8^+^ T cell infiltration was markedly greater in tumors from *Nat10*^cKO^ mice (Figure 2D and Supplemental Figure 2D). Using classical markers, we further classified CD8^+^ T cells into 5 distinct subsets: exhausted T cells, effector T cells, tissue-resident memory T cells, naive T cells, and memory T cells (Figure 2E). Notably, the proportion of effector T cells was substantially elevated and that of exhausted T cells was reduced in tumors from *Nat10*^cKO^ mice (Figure 2F), accompanied by enhanced CD4^+^ and CD8^+^ T cell infiltration (Figure 2, G and H) and elevated GzmB and IFN-γ production by CD8^+^ T cells (Figure 2I).

To validate the correlation between NAT10 expression and immune responses in human CRC, an analysis of patient cohorts revealed an inverse correlation between NAT10 expression and immune scores, activated CD8^+^ T cell infiltration, and effector memory CD8^+^ T cell infiltration (Figure 2J and Supplemental Figure 3, A–D). Moreover, single-cell data from tumor tissues of patients with clinical CRC receiving anti–PD-1 therapy demonstrated that responders exhibited lower NAT10 expression levels (Figure 2K). Furthermore, multiplex immunohistochemical staining of primary human CRC tissue microarrays confirmed negative correlation between NAT10 protein expression and CD8^+^ T cell infiltration (Figure 2, L and M), with high NAT10 protein expression and low CD8^+^ T cell infiltration predicting poor patient survival (Figure 2N). Using CellChat, an algorithm for mapping cell-cell communications within scRNA-seq datasets, we demonstrated enhanced ligand-receptor interactions between *Nat10*-KO tumor cells and TME components, particularly CD8^+^ T cells (Supplemental Figure 3, E–G).

Furthermore, immunocompetent mice bearing MC38 *Nat10*-KO tumors were treated with neutralizing antibodies to eliminate CD8^+^ or CD4^+^ T cells (Supplemental Figure 4A), which markedly reduced the respective abundances of CD8^+^ and CD4^+^ T cells in the spleen (Supplemental Figure 4B). Depletion of CD8^+^ T cells markedly reversed *Nat10*-KO–mediated tumor suppression (Supplemental Figure 4, C–E), whereas CD4^+^ T cell or macrophage depletion showed no effect (Supplemental Figure 4, C–J). Collectively, these findings establish intestinal Nat10 as a regulator of CD8^+^ T cell–dependent antitumor immunity, with its ablation creating an immunogenic TME that restricts CRC progression.

### NAT10 deficiency in tumor cells increases the infiltration of CD8^+^ T cells and enhances their cytotoxic functions.

**Figure 3:**
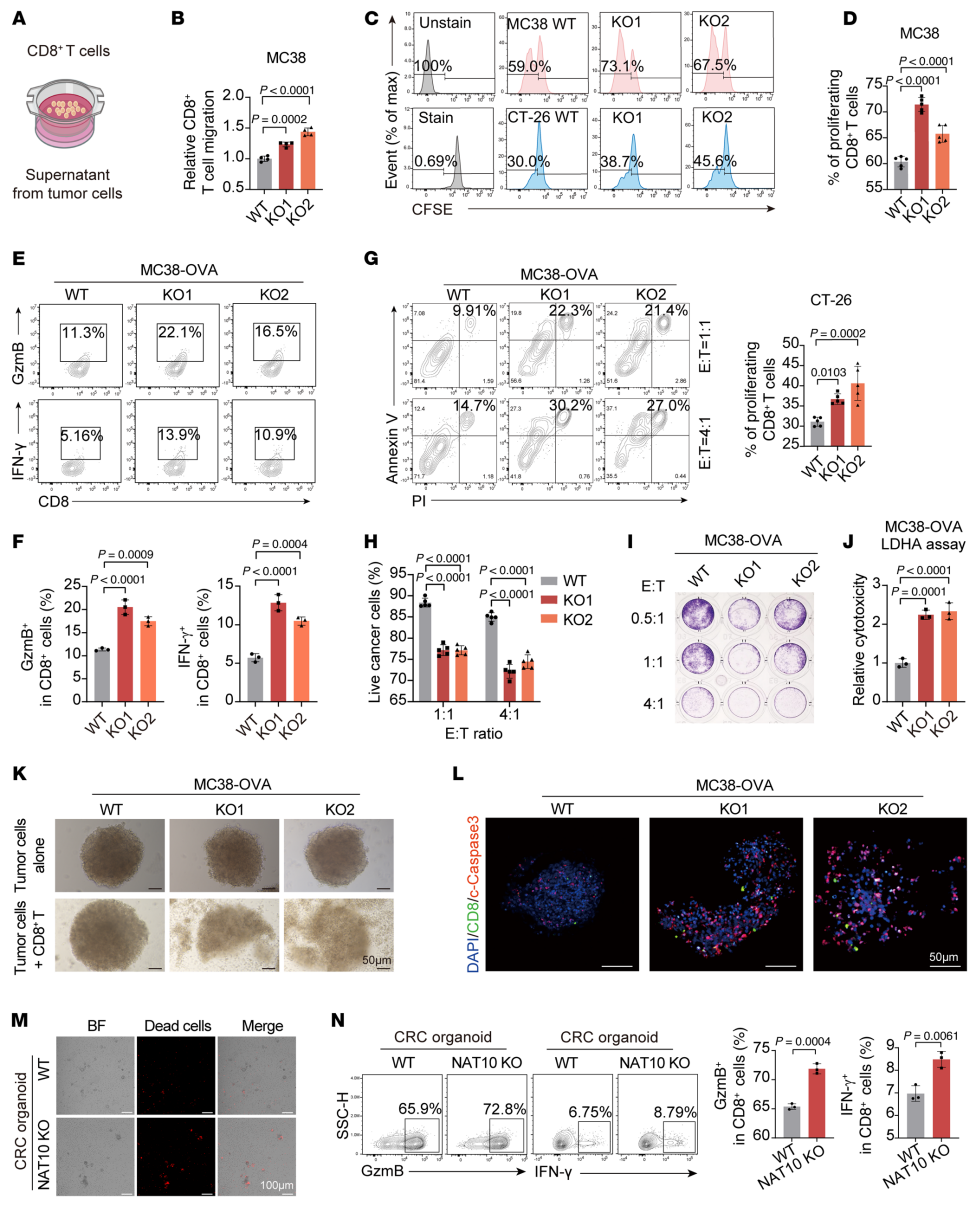
*NAT10* deficiency in tumor cells enhances CD8^+^ T cell infiltration and cytotoxic functions. (**A**) Schematic of the in vitro CD8^+^ T cell migration assay. (**B**) Flow cytometric analysis of CD8^+^ T cell migration toward conditioned media from MC38 WT/*Nat10*-KO tumor cells. (**C** and **D**) Flow cytometric analysis of CFSE-labeled CD8^+^ T cell proliferation following 72-hour coculture with WT/*Nat10*-KO tumor cells at a 1:1 ratio. (**E**–**J**) OT-1 CD8^+^ T cells cocultured with OVA-modified WT/*Nat10*-KO tumor cells at different E:T ratios for 24 hours. (**E** and **F**) Flow cytometry of GzmB^+^/IFN-γ^+^ CD8^+^ T cells. (**G** and **H**) Tumor cell apoptosis and OT-1 CD8^+^ T cell cytotoxicity were assessed by (**I**) crystal violet staining and (**J**) LDHA release assay. (**K** and **L**) OT-1 CD8^+^ T cells cocultured with OVA-modified MC38 WT/*Nat10*-KO tumor cells (1:1, 24 hours) in a 3D coculture system. (**K**) Representative images of tumor spheroid disintegration and (**L**) mIHC staining (apoptotic tumor cells: cleaved caspase-3, red; CD8^+^ T cell infiltration, green). Scale bar: 50 μm. (**M** and **N**) Human CRC organoids cocultured with autologous peripheral blood–derived CD8^+^ T cells. Representative fluorescence images of apoptotic cells in WT/*NAT10*-KO organoids (**M**). Scale bar: 100 μm. Flow cytometry of GzmB^+^/IFN-γ^+^ CD8^+^ T cells (**N**) (*n* = 3). The data are presented as the mean ± SD of 3 independent experiments (**B**, **D**, **F**, **H**, **J**, and **N**). One of 3 representative experiments is shown (**I** and **K**–**M**). Statistical analysis was performed by 1-way ANOVA (**B**, **D**, **F**, **H**, and **J**) and 2-tailed Student’s *t* test (**N**). ns, *P* ≥ 0.05. *P* < 0.05 was considered to indicate statistical significance.

Given the observed enrichment of cytotoxic CD8^+^ T cells in *Nat10*-deficient tumors, we investigated direct tumor-T cell interactions using in vitro models. Using an in vitro T cell migration assay, we confirmed that knockdown of *Nat10* in murine MC38 and CT-26 cells enhanced CD8^+^ T cell chemotaxis, whereas overexpression of *Nat10* in MC38 cells suppressed the migratory capacity of these cells (Figure 3, A and B, and Supplemental Figure 5, A–C). Flow cytometric analysis of tumor–T cell cocultures revealed that, compared with their *Nat10*-KO counterparts, WT tumor cells markedly inhibited CD8^+^ T cell proliferation (Figure 3, C and D). Next, we established an in vitro coculture assay involving OVA-specific OT1 CD8^+^ T cells (isolated from OT1 transgenic mice) cocultured with OVA-modified tumor cells to evaluate the effect of WT or *Nat10*-KO tumor cells on the cytotoxic function of CD8^+^ T cells. OT1 CD8^+^ T cells produced substantially higher levels of GzmB and IFN-γ when cocultured with *Nat10*-KO MC38-OVA/CT-26-OVA cells compared with OVA-WT counterparts (Figure 3, E and F, and Supplemental Figure 5, D and E). This phenotype was also observed in melanoma cell models, where *Nat10*-KO B16F10-OVA cells exhibited a milder suppression on CD8^+^ T cell cytotoxicity (Supplemental Figure 5, F–H). Moreover, quantitative cytotoxic T lymphocyte assays demonstrated that the proportion of apoptotic *Nat10*-KO MC38-OVA cells increased across different effector-to-target ratios (Figure 3, G–I). Correspondingly, *Nat10* depletion promoted the lactate dehydrogenase A (LDHA) release in OVA-WT tumor cells cocultured with OT1 CD8^+^ T cells (Figure 3J and Supplemental Figure 5I). Furthermore, 3D coculture systems revealed that *Nat10*-KO–OVA tumor spheroids exhibited a lower structural integrity and a higher caspase-3 activation compared with WT-OVA controls (Figure 3, K and L, and Supplemental Figure 5, J and K). Additionally, coculture of human CRC organoids with autologous CD8^+^ T cells showed that NAT10-KO organoids enhanced the killing capacity and cytotoxic function of CD8^+^ T cells (Figure 3, M and N, and Supplemental Figure 5, L and N). These data establish NAT10 as a tumor-intrinsic regulator that constrains recruitment of CD8^+^ T cell and impairs their cytotoxic function across multiple cancer models.

### DKK2 mRNA is a direct target of NAT10-mediated ac4C modification.

**Figure 4:**
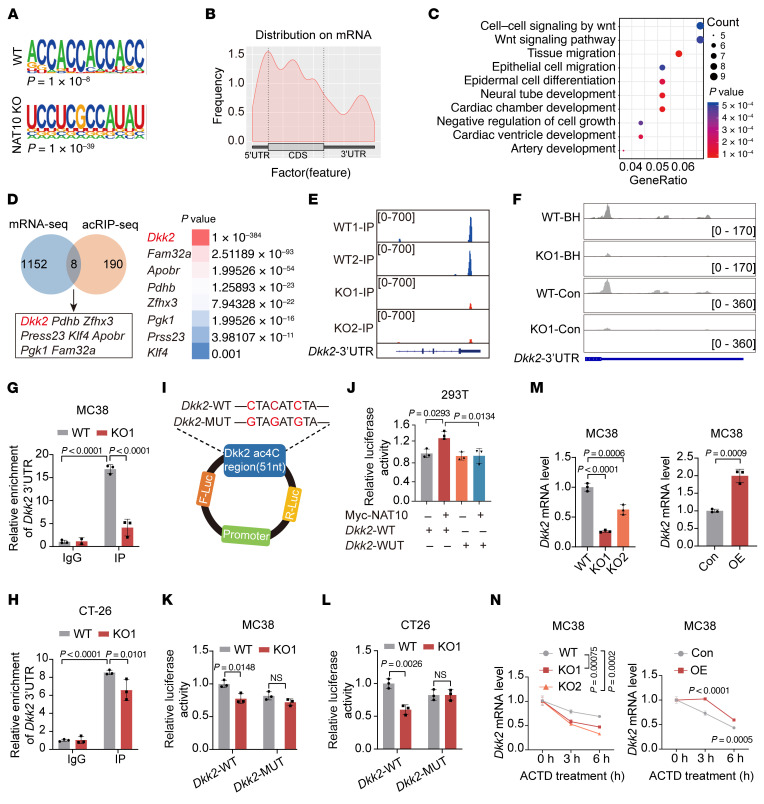
NAT10 directly targets *DKK2* mRNA for ac4C modification to stabilize its expression. (**A**) Consensus motif analysis of ac4C-modified transcripts by HOMER in MC38 WT/*Nat10*-KO cells. (**B**) Metagene plot showing the distribution of ac4C-containing peaks across mRNAs. (**C**) Gene ontology (GO) analysis highlighting pathways significantly enriched in ac4C-modified genes. (**D**) Venn diagram of candidate genes with altered ac4C peaks (acRIP-seq) and transcript levels (mRNA-seq) upon *Nat10* KO. *Dkk2* exhibited the most pronounced reduction in ac4C modification. (**E**) Integrative Genomics Viewer (IGV) tracks of ac4C peak on *Dkk2* mRNA based on acRIP-seq data in both MC38 WT/*Nat10*-KO cells. (**F**) IGV diagrams displaying read distributions and chemical ac4C sequencing–derived ac4C altered abundance across the 3′-UTR of DKK2 mRNA. (**G** and **H**) acRIP-qPCR quantification of *Dkk2* mRNA using anti-ac4C in WT/*Nat10*-KO cells. (**I**) Schematic of the dual-luciferase reporter containing the WT and mutant (MUT) *Dkk2* ac4C motif. (**J**) 293T cells were cotransfected with Myc-*Nat10* plasmid and *Dkk2* WT or MUT reporters for 48 hours. Luciferase activity in each group was detected. (**K** and **L**) Luciferase activity of WT/MUT reporters in *Nat10*-KO versus control cells. (**M**) qRT–PCR analysis of *Dkk2* mRNA levels in *Nat10*-KO/*Nat10*-overexpressing (OE) CRC cells. (**N**) qRT–PCR analysis of *Dkk2* mRNA levels in MC38 *Nat10*-KO/OE cells treated with actinomycin D (2.5 μg/mL) at the indicated time points. The data are presented as the mean ± SD of 3 independent experiments (**G**, **H**, and **J**–**N**). Statistical analysis was performed by 2-way ANOVA (**G**, **H**, **J**, and **N**), 1-way ANOVA (**M**, left), and 2-tailed Student’s *t* test (**K**, **L**, and **M**, right). ns, *P* ≥ 0.05. *P* < 0.05 was considered to indicate statistical significance.

To further explore the molecular mechanism by which Nat10 regulates antitumor immunity, ac4C RNA immunoprecipitation sequencing (acRIP-seq) and RNA-seq were conducted in *Nat10*-deficient MC38 cells and control cells. Sequence motif analysis revealed enrichment of “CxxCxxCxx” patterns at ac4C-modified sites (Figure 4A), with ac4C peaks predominantly localized in coding sequences and 3′-UTRs (Figure 4B and Supplemental Figure 6A). Gene ontology analysis further revealed that ac4C-modified genes were substantially enriched in the Wnt signaling pathway (Figure 4C). Integration of RNA-seq and acRIP-seq data identified 8 candidate genes exhibiting both ac4C modification and reduced expression upon *Nat10* KO, with the Wnt antagonist *Dkk2* displaying the most pronounced ac4C peak (Figure 4D). Furthermore, Integrated Genomics Viewer (IGV) visualization showed that the ac4C peaks were distributed in the 3′-UTR of *Dkk2* mRNA in WT cells but were diminished in *Nat10*-KO cells (Figure 4E). Since acRIP-seq may overestimate the prevalence of ac4C modification on mRNAs, we further employed NaCNBH_3_-based chemical ac4C sequencing (ac4C-seq) for a rigorous validation. IGV visualization confirmed that ac4C modifications identified via chemical sequencing were also enriched in the 3′-UTR of the *Dkk2* transcript in WT cells but were absent in *Nat10*-KO cells (Figure 4F). Moreover, nucleotide-resolution mapping identified ac4C sites showing NaCNBH_3_-dependent misincorporation exclusively in WT samples (Supplemental Figure 6B).

Moreover, ac4C-RNA immunoprecipitation-quantitative PCR (acRIP-qPCR) confirmed reduced ac4C enrichment on *Dkk2* mRNA in *Nat10*-KO MC38 and CT-26 cells compared with WT controls (Figure 4, G and H). Moreover, dual-luciferase reporter assays demonstrated that Nat10 overexpression increased WT *Dkk2* 3′-UTR-driven luciferase activity but had no effect on ac4C-motif mutants (MUTs) (Figure 4, I and J). Conversely, *Nat10*-KO markedly decreased the luciferase activity of the WT reporter gene, whereas it had no effect on the MUT reporter gene (Figure 4, K and L). Additionally, *Dkk2* mRNA levels were decreased in the *Nat10*-KO cells and increased in *Nat10*-overexpression cells (Figure 4M and Supplemental Figure 6C). Given that the enrichment of the ac4C peak in the 3′-UTR of *Dkk2* mRNA was markedly reduced in *Nat10*-KO cells, we hypothesized that Nat10 regulates *Dkk2* mRNA stability. Thus, CRC cells were treated with actinomycin D (2.5 μg/mL) to examine RNA decay, and the stability of *Dkk2* mRNA was markedly reduced in *Nat10*-KO cells, whereas the opposite effect was observed in cells overexpressing *Nat10* (Figure 4N and Supplemental Figure 6D). Therefore, we identified *Dkk2* as a direct target of Nat10, whose mRNA stability is regulated through ac4C modification in the 3′-UTR.

### NAT10 modulates CD8^+^ T cell recruitment and cytotoxicity through DKK2 regulation.

**Figure 5:**
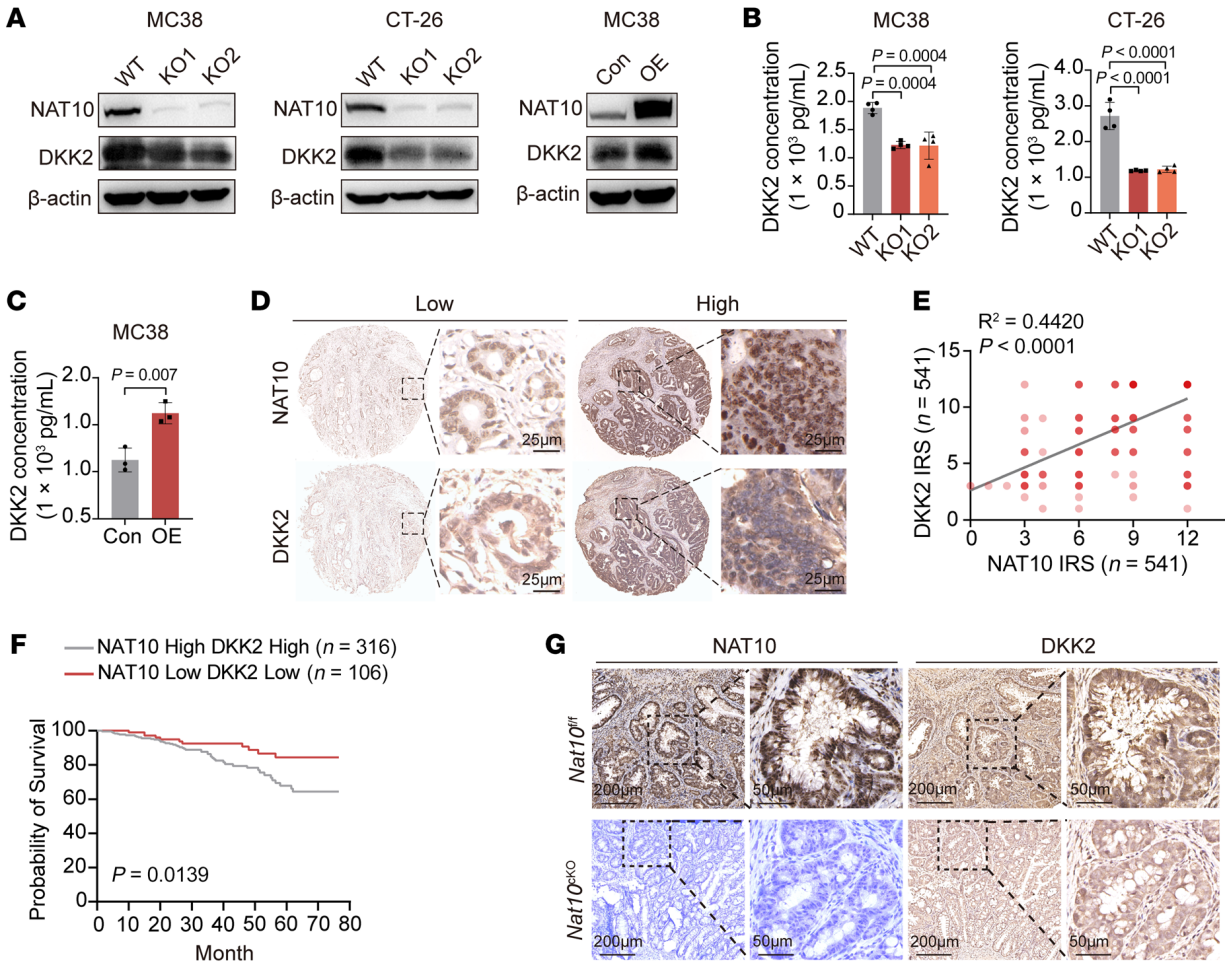
NAT10 positively regulates DKK2 expression with high expression of both associated with poor prognosis in patients with CRC. (**A**) Western blot analysis of Dkk2 protein levels in *Nat10*-KO/OE murine CRC cells. (**B** and **C**) ELISA quantification of secreted DKK2 in conditioned media from *Nat10*-KO/OE cells. (**D**) Representative IHC staining of NAT10 and DKK2 in human CRC tissues (*n* = 541). Scale bar: 25 μm; enlargement: original magnification, x40. (**E**) Positive correlation between NAT10 and DKK2 expression in CRC samples (*n* = 541). (**F**) Kaplan-Meier survival analysis of patients with CRC stratified by high versus low NAT10/DKK2 expression. (**G**) Representative IHC staining of Nat10 and Dkk2 expression in AOM/DSS-induced tumors from *Nat10*^fl/fl^ and *Nat10*^cKO^ mice (n = 5 mice/group). (**B** and **C**) The data are presented as the mean ± SD of 3 independent experiments. Statistical analysis was performed by 1-way ANOVA (**B**), 2-tailed Student’s *t* test (**C**), Pearson’s correlation coefficient (**E**), and log-rank test (**F**). ns, *P* ≥ 0.05. *P* < 0.05 was considered to indicate statistical significance.

To further investigate the NAT10-DKK2 regulatory axis, we analyzed DKK2 protein expression following genetic manipulation of NAT10. *Nat10*-KO cells exhibited markedly reduced DKK2 levels, whereas *Nat10* overexpression increased DKK2 levels (Figure 5A and Supplemental Figure 7A). Consistently, DKK2 expression decreased in *NAT10*-depleted human HCT116 cells but increased in *NAT10*-overexpressing SW620 cells (Supplemental Figure 7B). Since DKK2 is secreted, we quantified its levels in tumor cell conditioned media (CM). *NAT10* deficiency markedly reduced DKK2 levels in the CM of mouse CRC cell lines (MC38 and CT-26) and the human HCT116 cell line (Figure 5B and Supplemental Figure 7D). Comparable results were obtained in B16F10 cells, while *Nat10* overexpression increased secretion (Figure 5C and Supplemental Figure 7, C and D). Clinical correlation analyses revealed a positive association between NAT10 and DKK2 protein levels in human CRC tissues, and patients with high expression of both NAT10 and DKK2 had poorer prognosis (Figure 5, D–F). Consistently, the expression of Dkk2 was decreased in the intestinal tumors from *Nat10*^cKO^ mice (Figure 5G and Supplemental Figure 7E).

**Figure 6:**
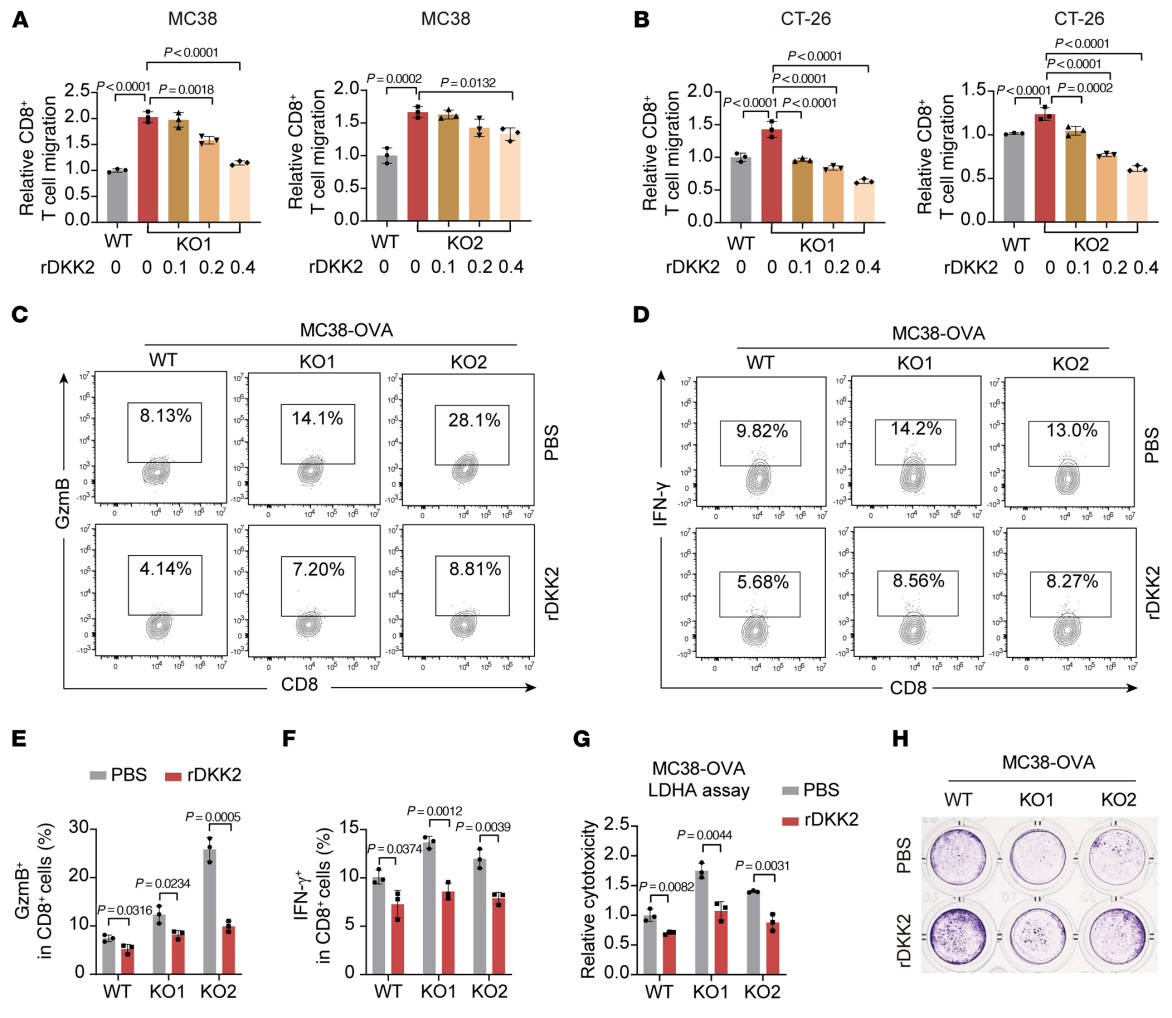
NAT10 orchestrates CD8^+^ T cell recruitment and cytotoxicity via aDKK2-dependent mechanism. (**A** and **B**) Flow cytometric analysis of CD8^+^ T cell migration toward conditioned media from WT/*Nat10*-KO tumor cells supplemented with or without recombinant Dkk2 (rDkk2) (μg/mL). (**C**–**F**) OT-1 CD8^+^ T cells were cocultured with OVA-modified MC38 WT/*Nat10*-KO tumor cells at 1:1 with or without rDkk2 for 24 hours. Flow cytometry analysis of GzmB^+^ and IFN-γ^+^ CD8^+^ T cell populations. (**G** and **H**) OT-1 CD8^+^ T cell cytotoxicity was assessed by LDHA release assay (**G**) and crystal violet staining assay (**H**). The data are presented as the mean ± SD of 3 independent experiments (**A**, **B**, and **E**–**G**). One of 3 representative experiments is shown (**H**). Statistical analysis was performed by 2-way ANOVA (**A** and **B**) and 2-tailed Student’s t test (**E**–**G**). ns, *P* ≥ 0.05. *P* < 0.05 was considered to indicate statistical significance.

Next, we investigated whether NAT10-driven DKK2 secretion suppresses antitumor immunity in vitro. Compared with WT cell control CM, *Nat10*-KO cell CM enhanced CD8^+^ T cell chemotaxis. Notably, this effect was abrogated by recombinant Dkk2 (rDkk2) supplementation in a dose-dependent manner. (Figure 6, A and B). We further explored whether Dkk2 directly impairs CD8^+^ T cell effector functions. OT1 CD8^+^ T cells were cocultured with OVA-expressing tumor cells (MC38, CT-26, and B16F10) in the presence or absence of rDkk2 (400 ng/mL). The results revealed that rDkk2 treatment markedly suppressed the production of GzmB and IFN-γ by CD8^+^ T cells cocultured with *Nat10*-KO cells (Figure 6, C–F, and Supplemental Figure 7, F–K). Additionally, the enhanced tumor-killing effect of CD8^+^ T cells under *Nat10*-KO conditions was abrogated by rDkk2 supplementation (Figure 6, G and H, and Supplemental Figure 7L). Collectively, these results suggest that NAT10 induces the secretion of DKK2 by tumor cells and that DKK2 serves as an immune suppressive factor that restricts the recruitment of CD8^+^ T cells and impairs their cytotoxic function in CRC.

### DKK2 promotes cholesterol biosynthesis to suppress CD8^+^ T cell antitumor function.

**Figure 7:**
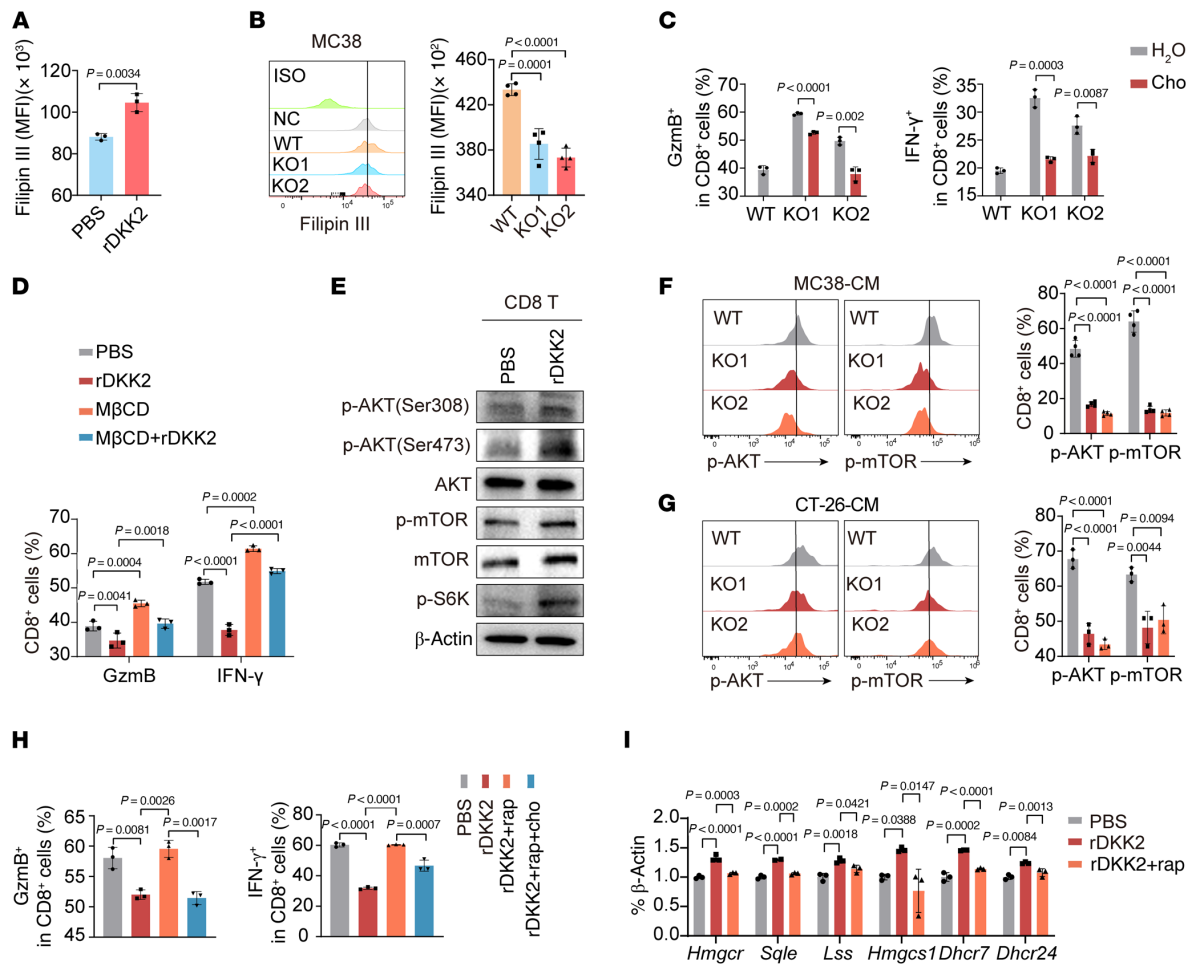
DKK2 promotes CD8^+^ T cell dysfunction via cholesterol accumulation. (**A**) Filipin III staining of cholesterol in CD8^+^ T cells with or without rDkk2 for 24 hours. (**B**) Cholesterol levels in activated CD8^+^ T cells cultured with MC38 WT/Nat10-KO cell conditioned media (CM) for 24 hours. (**C**) Flow cytometry of GzmB^+^/IFN-γ^+^ CD8^+^ T cell with cholesterol-supplemented tumor CM for 24 hours. (**D**) Flow cytometry of GzmB^+^/IFN-γ^+^ CD8^+^ T cell populations after 24 hours of culture with rDkk2 and MβCD. (**E**) Western blot of p-AKT/AKT, p-mTOR/mTOR, and p-S6K levels in CD8^+^ T cells. (**F** and **G**) Flow cytometry of p-AKT/p-mTOR levels in CD8^+^ T cells cultured with tumor CM for 24 hours. (**H**) Flow cytometry of GzmB^+^/IFN-γ^+^ CD8^+^ T cell populations treated with rDkk2 plus rapamycin or cholesterol. (**I**) qRT-PCR of cholesterol-related genes in activated CD8^+^ T cells after treatment with rDkk2 and rapamycin. The data are presented as the mean ± SD of 3 independent experiments (**A**–**D** and **F**–**I**). Statistical analysis was performed by 2-tailed Student’s *t* test (**A** and **C**), 1-way ANOVA (**B**, **F**, **G**, and **I**), and 2-way ANOVA (**D** and **H**). ns, *P* ≥ 0.05. *P* < 0.05 was considered to indicate statistical significance.

To elucidate the mechanism underlying the tumor-derived DKK2-mediated impairment of CD8^+^ T cell cytotoxicity, we investigated whether DKK2, a canonical Wnt signaling inhibitor (18), induced CD8^+^ T cell dysfunction by suppressing this pathway. Using SOST, a competitive Wnt antagonist (19), as a control, we found that SOST alone neither reduced GzmB and INF-γ secretion nor rescued rDkk2-induced CD8^+^ T cell dysfunction (Supplemental Figure 8, A and B). Transcriptomic profiling of CD8^+^ T cells exposed to rDkk2 revealed significant enrichment of pathways related to PPAR signaling, lipid digestion and absorption, and cholesterol metabolism (Supplemental Figure 8C). scRNA-seq data further revealed diminished cholesterol accumulation in tumor-infiltrating CD8^+^ T cells from *Nat10*^cKO^ mice versus *Nat10*^fl/fl^ controls (Supplemental Figure 8D), implicating Dkk2 in modulating CD8^+^ T cell cholesterol homeostasis. Intriguingly, we observed increased cholesterol content in CD8^+^ T cells treated with rDkk2 compared with control cells (Figure 7A). This finding is consistent with the established roles of cholesterol overload in T cell exhaustion (13, 20). Conversely, CD8^+^ T cells cultured in *Nat10*-KO tumor cell CM exhibited reduced cholesterol accumulation (Figure 7B and Supplemental Figure 8, E and F). Moreover, cholesterol supplementation in *Nat10*-KO CM suppressed GzmB and IFN-γ production by CD8^+^ T cells (Figure 7C and Supplemental Figure 8, G and H), whereas cholesterol depletion in CD8^+^ T cells via methyl-β-cyclodextrin (MβCD) (13, 21) increased GzmB and IFN-γ production and ameliorated rDkk2-induced cytotoxicity impairment (Figure 7D).

**Figure 8:**
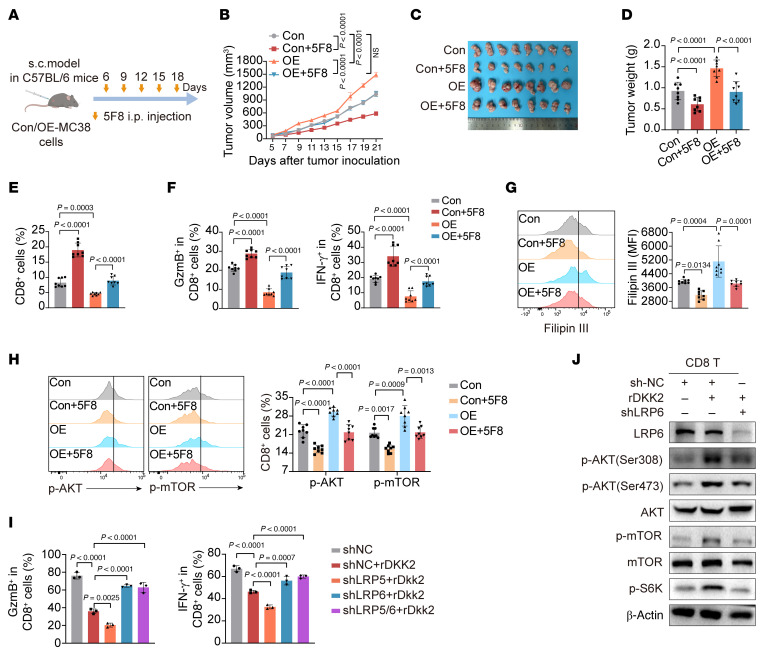
NAT10 drives CD8^+^ T cell dysfunction via DKK2/LRP6/AKT-mTOR axis–mediated cholesterol accumulation. (**A**) Schematic of anti-DKK2 antibody (5F8) treatment in MC38 control (Con) or *Nat10*-OE cell-bearing tumor mice. (**B**–**D**) Tumor growth curves (**B**) (mean ± SEM), representative tumor images (**C**), and tumor weights (**D**) (*n* = 8 mice/group). (**E** and **F**) Flow cytometry of tumor-infiltrating CD8^+^ T cells (**E**) and GzmB^+^/IFN-γ^+^ CD8^+^ T cell populations (**F**) in tumors (*n* = 8 mice/group). (**G** and **H**) Filipin III staining of cholesterol (**G**) and flow cytometry of p-AKT/p-mTOR levels (**H**) in tumor-infiltrating CD8^+^ T cells (*n* = 8 mice/group). (**I**) Flow cytometry of GzmB^+^/IFN-γ^+^ CD8^+^ T cells treated with rDkk2 after *Lrp5* or *Lrp6* knockdown. (**J**) Western blot of p-AKT/AKT, p-mTOR/mTOR, and p-S6K expression in *Lrp6*-knockdown CD8^+^ T cells. Data are shown as the mean ± SD of per group (**B**–**H**). The data are presented as the mean ± SD of 3 independent experiments (**I**). All statistical analysis was performed by 2-way ANOVA. ns, *P* ≥ 0.05. *P* < 0.05 was considered to indicate statistical significance.

Mechanistically, given the established link between AKT-mTOR signaling and cholesterol biosynthesis (20, 22), we observed rDkk2-induced activation of AKT-mTOR-S6K signaling in activated CD8^+^ T cells (Figure 7E). This pathway was activated in WT tumor cell CM-cultured CD8^+^ T cells but inhibited in *Nat10*-KO tumor cell CM-cultured ones (Figure 7, F and G, and Supplemental Figure 8I). In addition, pharmacological mTOR inhibition (rapamycin, 10 nM) rescued rDkk2-impaired cytotoxicity in CD8^+^ T cells (Figure 7H) and downregulated cholesterol biosynthesis-related genes (Figure 7I and Supplemental Figure 9A). However, exogenous cholesterol supplementation abrogated rapamycin-mediated restoration of CD8^+^ T cell cytotoxicity (Figure 7H). Furthermore, in vivo data showed that anti-Dkk2 antibody (5F8) not only inhibited tumor proliferation and growth (Figure 8, A–D, and Supplemental Figure 9B), but also notably reversed the acceleration of *Nat10*-OE tumor growth (Figure 8, B–D, and Supplemental Figure 9B). Meanwhile, 5F8 treatment substantially increased the numbers of tumor-infiltrating CD8^+^ T cells and effector T cells in *Nat10*-OE tumors (Figure 8, E and F). Additionally, 5F8 markedly reversed the elevation in the cholesterol levels and enhanced AKT-mTOR signaling activation in these infiltrating CD8^+^ T cells (Figure 8, G and H, and Supplemental Figure 9C). These results indicate that NAT10 promotes cholesterol accumulation in CD8^+^ T cells via DKK2 to impair their cytotoxicity.

As DKK2 binds to LRP5/6 to modulate glucose uptake and mTOR activation (22, 23), we next investigated receptor specificity. rDkk2 further exacerbated the reduction in GzmB and IFN-γ expression in *Lrp5*-knockdown CD8^+^ T cells, while knockdown of *Lrp6* in CD8^+^ T cells reversed the rDkk2-induced suppression of cytotoxicity (Figure 8I and Supplemental Figure 9D). Interestingly, dual *Lrp5*/*6* knockdown in CD8^+^ T cells partially restored cytotoxicity (Figure 8I), suggesting that LRP6 plays an important role in DKK2-mediated signaling. Moreover, DKK2-mediated activation of AKT-mTOR signaling in CD8^+^ T cells was alleviated by LRP6 knockdown (Figure 8J). Collectively, DKK2 engages LRP6 to hyperactivate AKT-mTOR-driven cholesterol biosynthesis, thereby suppressing the metabolism of CD8^+^ T cells in the TME.

### Targeting NAT10 or DKK2 augments anti-PD1 therapy to suppress CRC growth.

**Figure 9:**
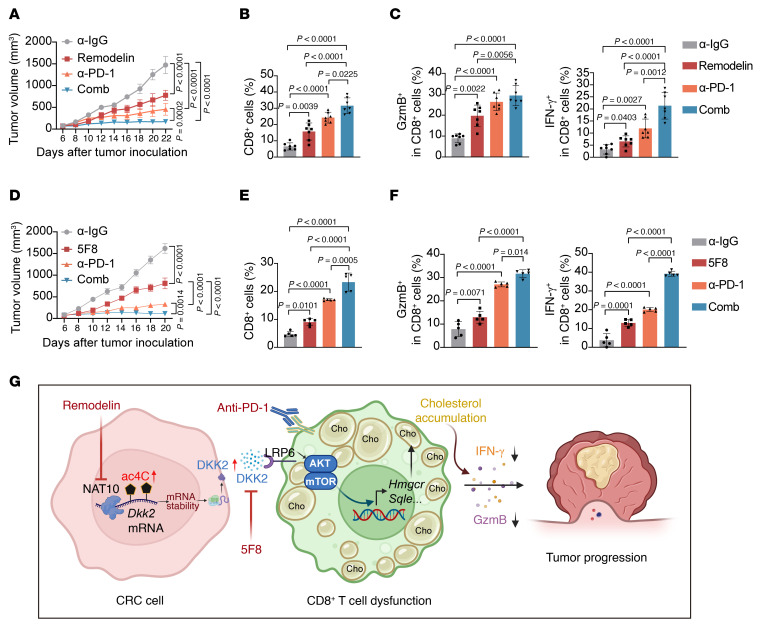
Dual targeting of NAT10 or DKK2 synergizes with PD1 blockade to suppress CRC. (**A**) Tumor growth curves. Data are shown as the mean ± SEM of 7 mice/group. (**B** and **C**) Flow cytometry analysis of tumor-infiltrating CD8^+^ T cells (**B**) and GzmB^+^ and IFN-γ^+^CD8^+^ T cell populations (**C**) in tumors form each group (*n* = 7 mice/group). (**D**) Tumor growth curves. Data are shown as the mean ± SEM of 7 mice/group. (**E** and **F**) Flow cytometry analysis of tumor-infiltrating CD8^+^ T cells (**E**) and GzmB^+^ and IFN-γ^+^CD8^+^ T cell populations (**F**) in tumors form each group (*n* = 5 mice/group). (**G**) Proposed model: tumor-intrinsic NAT10 stabilizes *DKK2* mRNA via ac4C modification, enabling DKK2-LRP6/AKT-mTOR signaling to drive cholesterol accumulation and dysfunction in CD8^+^ T cells, thereby promoting CRC progression. Dual targeting of NAT10 or DKK2 synergizes with anti–PD-1 therapy to suppress CRC. Data are shown as the mean ± SD of indicated mice per group. All statistical analysis was performed by 2-way ANOVA. ns, *P* ≥ 0.05. *P* < 0.05 was considered to indicate statistical significance.

Given the role of NAT10 in driving DKK2 secretion to suppress CD8^+^ T cell function within the CRC microenvironment, we evaluated whether inhibiting NAT10 or neutralizing DKK2 could potentiate immune checkpoint blockade (ICB) efficacy. In C57BL/6 mice bearing MC38 syngeneic tumors, Remodelin (5 mg/kg, intraperitoneal injection every 2 days), a selective NAT10 inhibitor, was combined with anti–PD-1 or IgG isotype control for 14 days (Supplemental Figure 10A). Compared with the control treatment, Remodelin monotherapy markedly attenuated tumor growth (Figure 9A and Supplemental Figure 10, B and C). Strikingly, Remodelin synergized with anti–PD-1 therapy, resulting in superior tumor growth suppression compared with either agent alone (Figure 9A and Supplemental Figure 10, B and C). Furthermore, Remodelin combined with anti–PD-1 treatment substantially increased the infiltration of CD8^+^ T cells into the tumor (Figure 9B), accompanied by a high percentage of GzmB^+^ and IFN-γ^+^ CD8^+^ T cells (Figure 9C), demonstrating that NAT10 inhibition enhances the effects of PD-1 blockade.

To further determine whether DKK2 neutralization similarly enhances ICB responsiveness, MC38 tumor-bearing mice were treated with anti-Dkk2 (5F8) alone or in combination with anti–PD-1 (Supplemental Figure 10D). Both 5F8 and anti–PD-1 monotherapies markedly reduced tumor burden (Figure 9D and Supplemental Figure 10, E and F), while their combination exhibited additive effects on tumor growth compared with the control or monotherapies (Figure 9D and Supplemental Figure 10, E and F). The dual-treatment group showed the greatest CD8^+^ T cell infiltration in MC38 tumors (Figure 9E) and the highest percentage of IFN-γ^+^GZMB^+^ CD8^+^ T cells (Figure 9F), mirroring NAT10-targeted outcomes. These findings indicate that disrupting the NAT10/DKK2 axis synergizes with PD-1 blockade to reinvigorate CD8^+^ T cell–mediated antitumor immunity, proposing a potential combinatorial immunotherapy strategy for CRC.

## Discussion

The ac4C writer NAT10 has been implicated in promoting malignant behaviors across multiple cancer types by modulating the ac4C modifications of various mRNAs or proteins, and its expression is strongly associated with tumor aggressiveness and poor clinical outcomes (24). While *NAT10* overexpression in CRC has been reported (16, 17), its role in shaping the immunosuppressive TME remains unexplored. Here, we reveal an unexpected immune-evasion mechanism, in which tumor-intrinsic NAT10 orchestrates CD8^+^ T cell dysfunction via the epigenetic regulation of DKK2. scRNA-seq and functional analyses in intestinal epithelium-specific NAT10-KO models revealed that NAT10 restricts CD8^+^ T cell infiltration and cytotoxicity, enabling immune evasion. Mechanistically, NAT10-mediated ac4C modification of DKK2 mRNA in tumor cells maintains DKK2 mRNA stability and promotes DKK2 secretion. Secreted DKK2 engages LRP6 on CD8^+^ T cells to activate the AKT-mTOR axis, reprogramming cholesterol metabolism and impairing effector function (Figure 9G). Our data demonstrate that NAT10 is a druggable epigenetic checkpoint in CRC immunotherapy.

Our syngeneic and carcinogen-induced CRC models demonstrated that Nat10 ablation enhances CD8^+^ T cell–dependent tumor control, as evidenced by increased cytotoxic CD8^+^ T cell infiltration, GzmB/IFN-γ production, and tumor regression. Furthermore, in vivo depletion experiments confirmed that the antitumor effects of NAT10 KO are primarily mediated by CD8^+^ T cells. In vitro studies further demonstrated that NAT10-deficient tumor cells promoted CD8^+^ T cell proliferation, migration and cytotoxicity. Our findings support NAT10 as a key regulator of CD8^+^ T cell–mediated immunosuppression in CRC.

To elucidate the molecular mechanism by which NAT10 promotes CRC, we integrated multi-omics analysis of acRIP-seq, NaCNBH3-based chemical ac4C-seq, and RNA-seq data to identify direct targets of NAT10, demonstrating *DKK2* as the pivotal NAT10 target. NAT10 directly binds to *DKK2* mRNA, inducing ac4C modification and increasing *DKK2* mRNA stability, leading to increased DKK2 expression. Consistent with these findings, we observed a positive correlation between the protein expression levels of DKK2 and NAT10 in both mouse and human CRC cell lines as well as in tumor tissues from patients with CRC and the AOM/DSS model. DKK2, a Wnt modulator, regulates immunity beyond the modulation of canonical β-catenin signaling (25). DKK2 is considered a critical negative regulator of Wnt/β-catenin signaling. It has been implicated in tumor cell survival, proliferation, migration, and invasion in various types of cancer (26, 27). While previous studies have linked DKK2 to angiogenesis and metastasis in CRC (28, 29), our work established that it plays an immunosuppressive role via metabolic reprogramming of CD8^+^ T cells. Furthermore, coculture experiments revealed that DKK2 suppresses CD8^+^ T cell migration and cytotoxicity, mirroring its inhibitory effects on NK cells (18, 29). Our study demonstrated that rDKK2 supplementation inhibited the migration and cytotoxic functions of CD8^+^ T cells, consistent with prior reports of DKK2-induced dysfunction in NK cells (18). Altogether, our data support the role of NAT10-driven DKK2 expression in suppressing CD8^+^ T cells and facilitating immune evasion in CRC.

CD8^+^ T cells are central to antitumor responses, but their function is often suppressed within TME. Restoring the cytotoxicity of CD8^+^ T cells is critical for effective cancer immunotherapy (30). Both intracellular and extracellular metabolic factors can contribute to CD8^+^ T cell dysfunction (30). Cholesterol, a crucial component of membrane lipids, is essential for T cell receptor (TCR) clustering and immune synapse formation and directly regulating T cell signaling and function (31–35). Aberrant cholesterol metabolism in T cells is implicated in various diseases, including cancer, infections, atherosclerosis, and autoimmune disorders (36–39). However, the role of cholesterol in the TME is complex. Previous studies have indicated that increased cholesterol levels in plasma membranes of CD8^+^ T cells promote TCR clustering and enhance antitumor responses (21), and other evidence suggests that cholesterol deficiency in intratumoral CD8^+^ T cells impedes their proliferation and survival (22). Conversely, some reports propose that cholesterol or cholesterol sulfate inhibits TCR signaling (40). The accumulation of cholesterol in tumor-infiltrating CD8^+^ T cells is associated with cellular exhaustion related to the upregulated expression of immune checkpoints (41). A recent study demonstrated that tumor-infiltrating CD8^+^ T cells exhibit higher cholesterol content than splenic T cells do and that abnormally increased cholesterol biosynthesis in CD8^+^ T cells triggers exhaustion and impairs their cytotoxic function (42). Moreover, tumor cells secrete cytokines such as FGF21 to promote cholesterol biosynthesis in CD8^+^ T cells, contributing to their dysfunction (42). Here, we found that NAT10 promotes DKK2 secretion by tumor cells, which increases cholesterol production and impairs the cytotoxic function of CD8^+^ T cells. Furthermore, we discovered that DKK2 activates the AKT-mTOR signaling axis to enhanced cholesterol biosynthesis in activated CD8^+^ T cells. Targeting this signaling cascade may normalize cholesterol levels and enhance CD8^+^ T cell–mediated antitumor immunity. These findings underscore the critical importance of regulating cholesterol metabolism in CD8^+^ T cells for effective antitumor immune responses.

Recent studies have shown that AKT-mTOR signaling is activated early after CD8^+^ T cell stimulation but gradually decreases after T cell activation and that sustained overactivation of mTOR signaling is detrimental to CD8^+^ effector T cells (20). Our results indicate that DKK2 maintains AKT-mTOR signaling in activated CD8^+^ T cells through LRP6, leading to cholesterol accumulation and impaired cytotoxicity. However, low-concentration rapamycin effectively inhibits AKT-mTOR signaling and restores CD8^+^ T cell function. In conclusion, we demonstrate that NAT10-mediated ac4C modification enables DKK2 to reshape the regulation of cholesterol metabolism by impairing CD8^+^ T cell functions and facilitating CRC progression.

Given the limited efficacy of ICB in microsatellite instability-high or mismatch repair-deficient patients with CRC, targeting the NAT10-DKK2 axis, which is involved in suppressing CD8^+^ T cell function may expand the application of ICB in CRC. Our work demonstrated that Remodelin, a specific inhibitor of NAT10, synergizes with anti–PD-1 therapy to inhibit CRC growth by restoring the cytotoxic function of GzmB^+^ and IFN-γ^+^ CD8^+^ T cells. Furthermore, DKK2, serving as a downstream target of NAT10, inhibits intratumoral CD8^+^ T cell infiltration and induces cholesterol accumulation in CD8^+^ T cells, impairing their cytotoxic function. Neutralizing DKK2 with the 5F8 enhances the efficacy of anti–PD-1 therapy by reactivating CD8^+^ T cells and inhibiting tumor growth. Unlike broad Wnt/β-catenin inhibitors, which may disrupt intestinal homeostasis, NAT10/DKK2 targeting offers precision by selectively neutralizing a CD8^+^ T cell–suppressive pathway.

In summary, we revealed the role of the ac4C writer NAT10 in shaping the immunosuppressive landscape of CRC. By coupling RNA epitranscriptomic regulation with immunometabolic crosstalk, we revealed that this pathway represents an actionable target to enhance ICB efficacy. Our findings support further exploration into the clinical application of NAT10 inhibitors or DKK2-neutralizing antibodies as adjuvants for PD-1 blockade, particularly in ICB-refractory CRC subsets.

## Methods

Additional details on methods can be found in the Supplemental Methods.

### Sex as a biological variable.

Both sexes were used for human and mouse studies. Sex was not considered as a biological variable.

### Mice.

LoxP-floxed *Nat10* (*Nat10*^fl/fl^) mice were generated by GemPharmatech, and Villin1-Cre (B6.Cg-Tg(Vil1-cre)997Gum/J) mice were acquired from The Jackson Laboratory. Intestinal epithelial cell–specific Nat10-deficient (*Nat10*^fl/fl^; Vil1-Cre^+^, namely *Nat10*^cKO^) mice were generated by crossing *Nat10*^fl/fl^ and Vil1-Cre^+^ mice. Genotyping of *Nat10*^cKO^ mice and WT mice was performed using PCR. The sequences of the primers used for PCR genotyping are listed in Supplemental Table 3. OT-I mice were purchased from Cyagen Biosciences. WT C57BL/6 mice, BALB/c mice, and BALB/c nude mice were purchased from GemPharmatech. All experimental mice were bred and maintained in a temperature-controlled room under a 12-hour-light/12-hour-dark diurnal cycle in a specific pathogen–free facility. All animal experiments were approved by the Institutional Animal Care Committee of Anhui Medical University.

### Statistics.

Statistical analyses were performed using GraphPad Prism 9.0 software. The data are presented as the mean ± SD unless otherwise indicated. Two-group comparisons were performed using 2-tailed Student’s *t* tests. One-way or 2-way ANOVA was used for comparisons among 3 or more groups with comparable variations. Survival estimates were obtained using the Kaplan-Meier method with a log-rank test. Correlation analysis of the immunohistochemistry immunoreactive score was performed using Pearson’s correlation coefficient. Each experiment was conducted with biological replicates and repeated no less than 3 times. Mice were randomly allocated to experimental groups. *P* < 0.05 was considered to indicate statistical significance.

### Study approval.

This study was approved by the Ethics Committee of the First Affiliated Hospital of Anhui Medical University (no. 2023047) and the Animal Experiments Committee of Anhui Medical University (no. 20230201). Written informed consent was obtained from all patients. The study was conducted according to the principles expressed in the Declaration of Helsinki.

### Data availability.

All the data during the current study are available within the paper and its supplemental materials or from the corresponding author upon reasonable request. Values for all data points in graphs are reported in the Supporting Data Values file. The raw sequence data reported in this paper have been deposited in the China National Center for Bioinformation/Beiiing Institute of Genomics, Chinese Academy of Sciences (GSA CRA022419) and are publicly accessible at https://ngdc.cncb.ac.cn/gsa Source data are provided with this paper.

## Author contributions

ML and SW designed the study. ML, XZ, JW, SZ, and YF performed the experiments. JW contributed to the bioinformatics analysis. C Chen and CY Chen cultured CRC organoids. YF, HW, XY, WD, and WZ provided the clinical samples. ML analyzed the clinical data. ZM, JX, and YQ helped with the in vitro experiments. ZW, BW, QW, QD, and WD commented on the study. ML and SW wrote the manuscript. SW supervised the research. All the authors read and approved the final manuscript.

## Funding support

- National Natural Science Foundation of China (U24A20720, 82073114, 82273157, 82372755, 82503474, 82102984, 82473107, and 82103266).
- China Postdoctoral Science Foundation (2024M760032).
- Natural Science Foundation of Anhui Province (2408085Y040 and 2408085QH235).

## Supplementary Material

Supplemental data

Unedited blot and gel images

Supplemental tables 1-4

Supporting data values

## Version 1 01/16/2026

Electronic publication
