# Supplementary material for: Targeting the N-acetyltransferase 10/DKK2 axis enhances CD8+ T cell antitumor activity in colorectal cancer models
Source: J Clin Invest. 2026 Jan 16;136(2):e196722. doi: 10.1172/JCI196722 (PMC12807479; doi:10.1172/JCI196722)
Supplement: Supplemental data [file jci-136-196722-s091.pdf]

Supplementary information

Supplementary Methods

Animal models

Nat10^{cKO} mice and wildtype littermates at 6-8 weeks of age were intraperitoneally injected with azoxymethane (AOM) (12 mg/kg body weight) (Sigma-Aldrich). One week later, the mice were administered drinking water supplemented with 2% dextran sulfate sodium (DSS) (Yeasen, Shanghai, China) for 7 days. DSS treatments were repeated for 3 cycles, and the mice were sacrificed at week 13.

Male BALB/c, C57BL/6 and BALB/c-nude mice, aged 5-6 weeks, were divided into groups for use in xenograft tumor models. *Nat10* knockout or control CT26 and MC38 cells (1 × 10^6/100 μL) were injected subcutaneously into the right ventral side of each mouse. The tumor volume was monitored every other day (volume = length × width^2 × 1/2). Two weeks after the injection, the mice were sacrificed and the xenograft tumors were dissected, and the weights of the tumors were measured.

For $CD8^+$ or $CD4^+$ T cell depletion, mice were implanted with 1 × 10^6 control or *Nat10*-KO MC38 tumor cells. An anti-CD8α antibody (clone 2.43; BioXCell, West Lebanon, New Hampshire, USA, BE0061) or an anti-CD4 (clone GK1.5; BioXCell, West Lebanon, New Hampshire, USA, BE0003-1) antibody was intraperitoneal injected on Days 0,4, 8,12 (200 μg in100 μL of PBS per mouse). For the control group, the same amount of isotype-matched rat IgG2b antibody

(clone LTF-2; BioXCell, West Lebanon, New Hampshire, USA, BE0090) was injected at the same time. For macrophage depletion, the mice were injected i.p. with 200 μL of clodronate liposomes or PBS liposomes (Yeasen, Shanghai, China, Cat# 40337ES) at 0, 4, 8, and 12 days after tumor inoculation.

For the therapeutic study, MC38 cells or MC38 Con/*Nat10*-OE were subcutaneously inoculated into the right flank of each mouse. Approximately 6 days after tumor cell inoculation, the mean volume was ≈100 mm^3, and the mice were injected with an isotype-matched rat IgG2a antibody (clone 2A3; BioXCell, West Lebanon, New Hampshire, USA, BE0089) as a control, Remodelin (10 mg/kg, intraperitoneally, every 2 days) (Selleck, Houston, USA, S7641), an anti–PD-1 antibody (150 μg/mouse, intraperitoneally, twice a week) (clone RMP1-14; BioXCell, West Lebanon, New Hampshire, USA, BE0146), an anti-DKK2 antibody (10 mg/kg, intraperitoneally, every 3 days). The mouse monoclonal antibody (mAb) to DKK2 (5F8) was generated via standard hybridoma technology by AbMax (Beijing, China), which involved immunizing mice with a synthetic peptide (KLNSIKSSLGGETPGC) of human DKK2.

Cell lines and cell culture

The HCT116 and SW480 CRC cell lines were purchased from the American Type Culture Collection (MD, USA), and the 293T, MC38, CT-26 and B16F10 cell lines were obtained from the Type Culture Collection of the Chinese Academy of Sciences (TCCCAS; Shanghai, China). CT-26, HCT116 and

SW480 CRC cells and $CD8^+$ T cells were cultured in RPMI-1640 medium (Invitrogen Life Technologies, CA, USA), and 293T, MC38 and B16F10 cells were cultured in DMEM (Invitrogen Life Technologies, CA, USA). The culture media were supplemented with 10% fetal bovine serum (FBS; Wisent, Montreal, Canada), 100 μg/mL streptomycin, and 100 U/mL penicillin (New Cell & Molecular Biotech, Suzhou, China), and all the cells were cultured in an incubator with 5% CO_2 at 37 °C. The cells were stored at -80 °C using CELLSAVING (New Cell & Molecular Biotech, Suzhou, China). All the cells tested negative for mycoplasma contamination and were authenticated via short tandem repeat (STR) profiling before use.

Human samples

CRC tissue microarrays, fresh tumor tissues and peripheral blood samples from CRC patients were obtained from the First Affiliated Hospital of Anhui Medical University. This study was approved by the Clinical Research Ethics Committees of the First Affiliated Hospital of Anhui Medical University and was conducted in accordance with the Helsinki Declaration ([2020] No. 052). All patients provided consent for sample collection. The clinicopathological characteristics of the clinical patients were included in Supplementary Table 4.

Establishment of organoid cultures

The organoid culture method was described in a previous study(1). Freshly

collected tissues were immediately placed into tissue storage solution (MACS). Approximately 1 cm^3 of tumor tissue from CRC patients was washed 2–3 times with 1× chelation buffer (containing 5.6 mM Na_2HPO_4, 8.0 mM KH_2PO_4, 96.2 mM NaCl, 1.6 mM KCl, 43.4 mM sucrose, 54.9 mM D-sorbitol, and 0.5 mM DL-dithiothreitol (DTT), pH = 7) supplemented with 10% penicillin-streptomycin, and then minced into small pieces of 2–3 mm^3. Subsequently, 1–2 mL of digestion solution (containing 5 mg/mL collagenase II, 50 μg/mL DNase I) was added, and the mixture was incubated in a shaker at 37°C for 15–20 min. After being digested, the tissues were filtered through a 70 mm filter and centrifuged at 1400 rpm for 5 min. Following washing, the cell precipitate was resuspended in Matrigel, and approximately 100 glands per 50 μL of Matrigel were seeded into each well of a preheated 24-well plate, which was then placed in a 37 °C incubator. Once the Matrigel solidified, 500 μL of complete CRC organoid medium (Mogengel, Xiamen, China) was added. The medium was supplemented every 3-4 days, and the organoids were passaged using TrypLE Express (Gibco) every 1-2 weeks.

To perform gene knockout in the organoids, Matrigel was digested with 1× Dispase II to collect CRC organoids. The organoids were further digested into single cells using TrypLE Express and counted. 2×10^5 cells were resuspended in medium containing polybrene (8 mg/mL) and inoculated with CRISPR-Cas9 gRNA lentivirus (500 x g, 1 h, 32 °C). After incubation at 37 °C for 6-8 h, the cells were resuspended in Matrigel and seeded into a 24-well plate. Puromycin

was introduced three days post-infection to isolate puromycin-resistant organoids, which were then confirmed by Western blotting.

For organoid sectioning, organoids were collected and fixed with 4% paraformaldehyde (PFA) at room temperature for 40 min. After fixation, the organoids were embedded in 3% agarose, followed by paraffin embedding, sectioning, and immunohistochemical staining.

T cells and organoid co-culture assays

$CD8^+$ T cells were isolated from peripheral blood by negative selection using the EasySep Direct Human $CD8^+$ T Cell Isolation Kit (Stemcell, Vancouver, Canada) and counted for T-cell activation experiments according to the manufacturer's instructions. A human CD3/CD28 T cell activator (Stemcell, Vancouver, Canada) was utilized to expand and activate the T cells. RPMI-1640 medium supplemented with 1% penicillin/streptomycin, 50 nM β-mercaptoethanol, 10 ng/mL IL-2 (Novoprotein, Suzhou, China) and 5ng/mL IL-7 (Novoprotein, Suzhou, China) was added every 3 days. After 4-5 days of expansion, the T cells were ready for co-culture experiments to assess the tumor killing efficacy.

Organoids intended for T-cell co-culture were removed from the solidified Matrigel using 1× dispase enzyme and resuspended in CRC organoid medium. To remove larger organoids, the suspension was filtered through a 100 mm cell strainer. Activated T cells were co-cultured with CRC organoids at a 5:1 ratio

for 24 h. The cells were placed in glass-bottom dishes for confocal microscopy (Corning), which contained 100 μL of T-cell medium and 100 μL of CRC organoid medium. NucRed Dead 647 (Thermo Fisher Scientific, Waltham, MA, USA, R37113) was added to label dead cells. Live imaging was performed 24 h post co-culture using a laser scanning confocal microscope (Olympus, FV3000). CRC organoids co-cultured with T cells were dissociated into single cells using TrypLE Express and incubated with CellEvent Caspase-3/7 Green (Thermo Fisher Scientific, Waltham, MA, USA, C10740) to label apoptotic cells and assess T-cell cytotoxicity. The co-cultured cell mixture was collected, resuspended in PBS, and stained with Fixable Viability Stain 780 (BD BioScience, New Jersey, USA) to label dead cells, then stained with APC-R700 mouse anti-human CD8 (BD BioScience, 566857), anti-human CD16 (Elabscience, E-AB-F1236A), PE mouse anti-human/mouse Granzyme B (BioLegend, 372207), and BV786 mouse anti-human IFN-γ (BD BioScience, 563731). After being washed twice with staining buffer, the cells were filtered through a 70 mm cell strainer and detected using a Full-Spectrum Multicolor Analytical Flow Cytometer (Cytek Aurora). Data analysis was performed via FlowJo software (FlowJo LLC).

Flow cytometry analysis of tumor-infiltrating immune cells

After the mice were euthanized, the tumors were immediately dissected. The tumors were minced and digested with 1640 (Gibco) supplemented with 5

mg/mL collagenase II (Roche, Basel, Switzerland) and 5 U/mL DNase I (Roche, Basel, Switzerland) on a 37 °C shaker at 180 rpm for 30 min. Complete medium containing 10% FBS was added to the mixture to terminate digestion. The mixture was filtered through a 70 μm cell strainer, washed once with PBS, and stained with Fixable Viability Stain 780 (BD BioScience, New Jersey, USA) for 10 min at room temperature in the dark. The cell mixture was washed once with PBS. The isolated cells were incubated with an anti-mouse CD16/32 antibody (Elabscience, E-AB-F0997A) on ice for 10 minutes to block nonspecific Fc receptor binding. The cells were subsequently incubated with the following antibodies on ice for 30 min: anti-CD45-BV605 (BD BioScience, 563053), anti-CD3-PE (BD BioScience, 553063), anti-CD8-FITC (BD BioScience, 553031), anti-CD4-BV510 (BD BioScience, 100449), anti-CD11b-PerCP (BioLegend, 101212), anti-F4/80-APC (BioLegend, 123116), anti-NK1.1-PE/Cyanine7 (BD BioScience, 562062), anti-Gr1-Alexa Fluor®700 (BioLegend, 108421). After washing, the cells were resuspended in FACS buffer, and a full-spectrum multicolor analytical flow cytometer (Cytek Aurora) was used to collect data. The data were analyzed via FlowJo software (FlowJo LLC).

For the intracellular staining of T-cell cytotoxic molecules, an activation cocktail (BD BioScience, New Jersey, USA) was used to stimulate intratumoral immune cells in vitro for 4-6 h. Subsequently, the cells were stained with Fixable Viability Stain 780 (BD Biosciences, 565388). Surface marker staining was performed using anti-CD45-BV605 (BD BioScience, 563053), anti-CD3-

PerCP/Cyanine5.5 (BioLegend, San Diego, California, USA), anti-CD3-APC (BioLegend, 100217), anti-CD8-FITC (BD BioScience, 553031), and anti-CD4-BV510 (BD BioScience, 100449). Then, fixation was performed for 20 min on ice using Fixation/Permeabilization Kit (BD BioScience, New Jersey, USA). The cells were then incubated for 30 minutes with anti-Granzyme B-PE (eBioscience, 12-8898-80) and anti-IFN-γ-PE/Cyanine7 (eBioscience, 25-7311-41). Intracellular staining for APC-T-bet (BioLegend, 644813) was also performed, and the cells were fixed and permeabilized using the Transcription Factor Buffer Set (BD BioScience, New Jersey, USA). Cells were washed with PBS, and data obtained by a full-spectrum multicolor analytical flow cytometer (Cytek Aurora). Gating strategies were indicated in Supplemental Figure 12.

Flow cytometry analysis of p-AKT and p-mTOR signaling

To stain for members of the p-AKT and p-mTOR signaling pathways in tumor-infiltrating $CD8^+$ T cells, the cells were incubated with the following antibodies: anti-CD45-BV605 (BD BioScience, 563053), anti-CD3-PE (BD BioScience, 553063) and anti-CD8-APC (Elabscience, E-AB-F1104E) antibodies as described above. After washed with PBS, the cells were fixed and permeabilized with the fixation buffer (BD BioScience, New Jersey, USA) and perm buffer III (BD BioScience, New Jersey, USA). The cells were stained with primary antibodies against BV421-Akt (pS473) (BD BioScience, 562599) and PE/Cy7-mTOR (pS2448) (eBioscience, 25-9718-41) on ice for 40 min. After

washed twice with PBS, the cells were resuspended in FACS buffer, and a full-spectrum multicolor analytical flow cytometer (Cytek Aurora) was used to acquire data. Gating strategies were indicated in Supplemental Figure 12.

Immunohistochemistry

For paraffin section, the tissue sections embedded in paraffin were dewaxed using xylene, followed by dewaxing in 100%, 90%, 80%, and 70% ethanol solutions. The dewaxed slides were then immersed in preheated citrate buffer for high-pressure antigen retrieval, cooled to room temperature, and soaked in PBS for 2 min. For frozen sections, the sections were incubated in a 37 °C oven for 1 h, and then immersed in PBS for 2 min. To reduce nonspecific background staining caused by endogenous peroxidase, the sections were incubated with an endogenous peroxidase blocker (Beyotime, Shanghai, China) for 15 min at room temperature and then washed three times with PBS. Subsequently, an immunostaining blocking solution (Beyotime, Shanghai, China) was applied for 30 min to block nonspecific background staining. The blocking solution was removed, and working solutions of the primary antibodies: anti-NAT10 (Abcam, ab194297), anti-DKK2 (Absin, abs102572), anti-Ki-67 (Abclone, A20018), and cleaced-Caspase3 (CST, 9664) were added to the slides, which were subsequently incubated overnight at 4 °C. The slides were then washed three times with TBST and incubated with rabbit-on-rodent HRP-Polymer (Beyotime, Shanghai, China) at room temperature for 30 min. After

three washes, DAB was used for chromogenic visualization, followed by scanning using a HALO Digital Pathology Image Analysis System (Indica Labs).

Multiplex immunohistochemistry (mIHC) analysis

Tissue sections embedded in paraffin were subjected to multiple IHC staining using the PANO 6-plex IHC Kit (Panovue, Beijing, China) according to the manufacturer's protocol as described previously(2). The following antibodies primary antibodies were applied to the sections sequentially: anti-CD8 (Abcam, ab217344), anti-CD8 (Proteintech, 66868-1-Ig), anti-CD4 (Abcam, ab183685), anti-CD4 (Proteintech, 67786-1-Ig), anti-NAT10 (Abcam, ab194297), anti-Granzyme B (Proteintech, 13588-1-AP), anti-CD68 (Abclone, A22329), anti-PD-1 (CST, 84651), anti-Tim3 (Abcam, ab241332), anti-F4/80 (Abclone, A23788), anti-Perforin (Abclone, A0093) and anti-LAG3 (Proteintech, 16616-1-AP). The sections were subsequently incubated with horseradish peroxidase-conjugated secondary antibodies and a tyramide signal amplification (TSA) working solution. After each TSA step, the slides were heated in a microwave, and multiple IHC procedures were carried out using the same protocol. After all the primary antigens were labeled, the sections were placed on slides and covers lipped with anti-fade mounting medium containing DAPI. The slides were scanned using a slid eview (Olympus, VS200), and 3 to 10 fields of view were captured based on the tissue size for multispectral imaging analysis.

Assessment of immunohistochemistry

Immunohistochemical staining results were independently scored by two pathologists blinded to clinical data using the semi-quantitative immunoreactive score (IRS), as previously reported (3). The optimum cutoff value for IRS was determined via receiver operating characteristic (ROC) analysis (4), the area under the curve (AUC) was calculated at different cutoff values of NAT10 or DKK2 IRS for 5 years of overall survival (OS) time, which is a key indicator for assessing the prognosis of cancer patients in clinic. The optimal value of cutoff points of the NAT10 or DKK2 IRS in CRC cohort was 6 due to the predictive value of this cutoff point for death was the best (Supplementary Figure11). Under these criteria, samples with IRS 0–6 and IRS 8–12 were classified as low expression and high expression, respectively. Selected the average value of the ratio of the number of $CD8^+$ T cells to the total number of cells in three different fields of view of each tumor tissue as the percentage of CD8-positive cells. The median value was used as the cutoff to stratify the percentage of $CD8^+$ T cell infiltration into two groups: high infiltration (≥5.3125%) and low infiltration (<5.3125%).

Lentiviral infection

Lentiviruses (Cas9-Puro)/U6-*Nat10*-KO and (Cas9-Puro)/-NC were constructed by Corues Biotechnology (Nanjing, China). KO1 and KO2 are *Nat10*-KO cells generated via the Clustered Regularly Interspaced Short

Palindromic Repeats/Cas9 (CRISPR/Cas9) gene editing system, using two distinct guide RNAs. The sgRNA sequences are listed in Supplementary Table 2. The *Nat10*-OE lentiviruses were produced by GeneChem Co., Ltd. (Shanghai, China) using GV341 vectors (Ubi-MCS-3FLAG-SV40-puromycin). The OVA lentiviral plasmid was constructed by YouBio (Changsha, China) using the pCDH-CMV-MCS-EF1a-RFP+BSD vector. The *Lrp5*/*Lrp6*-knockdown lentiviral plasmid was constructed by Hanbio (Shanghai, China) using the HBhTLV-EF1-ZsGreen-Puro vector. The sequences are listed in Supplementary Table 2.

In brief, tumor cells were infected with lentivirus for 48-72 h. Stably knockout or stably expressing tumor cell lines were selected with puromycin (Sigma, St. Louis, Missouri, USA) or Blasticidin (Sigma, St. Louis, Missouri, USA). To generate *Nat10*-knockout (*Nat10*-KO) cells, single cells were then distributed into 96-well plates, and the *Nat10*-KO clones were validated by immunoblotting.

For T cells infected with lentivirus, T cells activated with Dynabeads™ Mouse T activator CD3/CD28 (Thermo Fisher Scientific, Waltham, MA, USA) for 24-48 h were spinduced shNC or sh*LRP5*/*LRP6* viral supernatant supplemented with polybrene at 300 × g, 35 °C for 1.5 h, and then placed in a 37 °C incubator. After 6-8 h, fresh complete medium was added. Between 48 and 72 h later, the transfected (ZsGreen$^+$ CD8$^+$) T cells were gated or sorted for experimentation and analysis.

Preparation of tumor-conditioned medium (TCM) preparation

Tumor-conditioned medium (TCM) was prepared as described previously(5, 6). In brief, tumor cells were seeded in 150 mm cell culture dishes. When the cells reached 80% confluence, the culture medium was removed, and the cells were washed three times with PBS. They were then cultured in serum-free medium for 48 h. Afterward, the conditioned medium was collected and filtered through a 0.22 mm filter, followed by storage at 80 °C until use.

T cell suppression assay

Splenic murine $CD8^+$ T cells were isolated from C57BL/6 or BLAB/c mice aged 6-10 weeks by using an EasySep mouse $CD8^+$ T cell isolation kit (Stemcell, Vancouver, Canada) according to the manufacturer's instructions. Then, the cells were labeled with CFSE (Invitrogen, Carlsbad, California, USA) at 1 μM for 10 min and resuspended in RPMI 1640 media supplemented with 10% FBS, 1% penicillin/streptomycin and 50 nM β-mercaptoethanol. CFSE-labeled T cells were co-cultured with tumor cells in 96-well plates at a ratio of 1:1 for 72 h in medium containing Dynabeads™ Mouse T activator CD3/CD28 (Thermo Fisher Scientific, Waltham, MA, USA) and IL-2 (Novoprotein, Suzhou, China). CFSE intensity were quantified by flow cytometry.

$CD8^+$ T migration assay

Migration of $CD8^+$ T cells was assessed in 24-well plates with 5.0 μm Transwell inserts. Activated 5×10^4 $CD8^+$ T cells were placed in the upper chamber of the Transwell system. CM from WT or *Nat10*-KO tumor cells were added in the bottom chamber of the Transwell. After incubation in a 37 °C incubator for 6 h, the cells in the upper chamber were removed, and the number of migrated $CD8^+$ T cells in the lower chamber was counted via flow cytometry.

T cell function analysis

Before the end of co-culture or the termination of tumor cell CM or drug treatment, the leukocyte activation cocktail (BD BioScience, New Jersey, USA) was added to stimulate the cells for 4-6 h. Subsequently, cells were stained with Fixable Viability Stain 780 (BD BioScience, New Jersey, USA). The samples were then washed and resuspended in staining buffer and stained with a FITC-conjugated anti-CD8a antibody (BD BioScience, 553031) for 30 min on ice. After washing with PBS, the cells were fixed for 20 min on ice using a Fixation/Permeabilization Kit (BD Biosciences, New Jersey, USA). The cells were then incubated in permeabilization buffer for 30 min with anti-Granzyme B-PE (BioLegend, 372207) and anti-IFN-γ-PE/Cyanine7 (BioLegend, 505825). Cells were washed and analyzed with a full-spectrum multicolor analytical flow cytometer (Cytek Aurora).

For TCM treatment, T cells were treated with TCM and complete medium (ratio 3:1). For other treatments, rDKK2 (400 ng/mL) (R&D, 2435-DKB-010), β-

cyclodextrin (MβCD, 1 mM) (Sigma, C4555), cholesterol (0.75 mg/mL) (Sigma, C4951), or rapamycin (10 nM) (MedChemExpress, New Jersey, USA) were added to T cells.

T cell killing assay

OT-1 $CD8^+$ T cells were isolated from the spleens of OT-1 mice using EasySep Mouse $CD8^+$ T Cell Isolation Kit (Stemcell, Vancouver, Canada). OT-1 T cells were activated with magnetic beads for 3 days. WT or *Nat10*-KO cells (MC38-OVA, CT26-OVA, and B16F10-OVA) were co-cultured with OT-1 $CD8^+$ T cells at different E: T ratios (0.5:1, 1:1, and 4:1) in 96-well plate with a total volume of 200 μL. Twenty-four hours later, the apoptotic tumor cells were detected using an Annexin V-FITC/PI Apoptosis Detection Kit (Vazyme, Nanjing, China), according to the manufacturer's protocol. T cells and cellular debris were removed via washes with PBS, followed by staining with crystal violet to assess the number of viable tumor cells. LDH release was determined using a lactate dehydrogenase assay kit (Jiancheng Bioengineering Institute, Nanjing, China) following the manufacturer's instructions.

3D co-culture system

The culture methods used were previously described (7, 8). $CD8^+$ T cells were isolated from the spleens of C57BL/6 or BALB/c mice and activated for 3 days *in vitro*. Activated $CD8^+$ T cells were mixed with murine MC38/CT-26 WT or

Nat10-KO cancer cell lines at a ratio of 1:1 and co-cultured in a 3D Petri® dish (Micro-Tissues, Rhode Island, USA) for 24 h. Cell spheroids were released from the microtissues, and their tumor cell spheroid-forming ability was assessed.

MC38/CT-26 WT or *Nat10*-KO cancer cell lines were cultured in 3D Petri dish (Micro-Tissues, Rhode Island, USA) for 24-48 h until 3D tumor spheroids formed. These spheroids were isolated from the microtissues and co-cultured with activated $CD8^+$ T cells at a 1:1 ratio for 24 h. The spheroids were subsequently fixed, embedded in paraffin, and analyzed by mIHC.

Filipin III staining of cholesterol

Filipin III was dissolved in ethanol to reach the final concentration of 5 mg/mL. The cells were fixed with 4% paraformaldehyde, stained with 50 μg/mL filipin III (MedChemExpress, New Jersey, USA) for 1 h at room temperature and analyzed via flow cytometry (BD FACSAria™ III). For staining of tissue sections, the sections were stained with 100 μg/mL Filipin III at room temperature for 1 h, followed by imaging using a confocal laser scanning microscope (Leica STELLARIS 5, Germany).

Enzyme-linked Immunosorbent Assay (ELISA)

WT and *NAT10*-KO tumor cells (2×10^6) were seeded into a 100 mm dishes in complete medium, and the supernatants were collected 48-72 h later. The secretion of DKK2 in the supernatants was measured by using mouse (JonIn,

Shanghai, China) or human ELISA kit (Cusabio, Wuhan, China) according to the manufacturer's instructions. The protein level was calculated on the basis of the standard curve.

RNA stability assay

Nat10-knockdown or *Nat10*-overexpressing CRC cells were treated with 2.5 μg/mL actinomycin D (MedChemExpress, New Jersey, USA) for 0, 3 or 6 h. Total RNA was subsequently isolated, and the relative levels of *Dkk2* were measured via qPCR. The primer sequences are listed in Supplementary Table 3.

RNA immunoprecipitation-qPCR (RIP-qPCR)

The PureBinding® RNA Immunoprecipitation Kit (Geneseed, Guangzhou, China) was used according to the manufacturer's instructions as previously described(1). Briefly, lysates from the indicated cells were incubated with magnetic beads coupled with either 5 μg of control IgG (Beyotime, A7016) or an anti-ac4C antibody (Abcam, ab252215) under rotation overnight at 4 °C. The RNA bound to the magnetic beads was subsequently extracted, and the expression of *Dkk2* was measured via qRT-PCR. The primer sequences for acRIP-qPCR are listed in Supplementary Table 3.

Luciferase reporter assay

The luciferase reporter vector (pGL-SV40-Rluc-TK-Luc) was constructed by Corues Biotechnology (Nanjing, China). The *Dkk2*-WT (51 nt) sequence used was CTGATTAAACACATTTCTGAATACTACATCTAGAGCATAGAGGACAAAAAT; the *Dkk2*-MUT (51 nt) sequence was CTGATTAAACACATTTCTGAATAGTAGATGTAGAGCATAGAGGACAAAAAT. Forty-eight hours post cell transfection, the relative luciferase activity was assessed using a dual-luciferase reporter assay system (Vazyme, Nanjing, China) following the manufacturer's protocol.

Western blot

Western blotting was performed as previously described(9, 10). The antibodies used were as follows: anti-AKT (CST), anti-phospho-Akt (Ser473) (Huabio), anti-phospho-Akt (Thr308) (CST), anti-mTOR (Huabio), anti-phospho-mTOR (Ser2448) (Affinity), anti-phospho-S6 Ribosomal Protein (Ser235/236) (CST), anti-DKK2 (Absin), anti-LRP5 (Proteintech), anti-LRP6 (ABclonal), anti-β-actin (Proteintech), and anti-GAPDH (Proteintech). The detailed information of antibodies is listed in Supplementary Table 1.

Quantitative RT-PCR (qRT–PCR)

Total RNA was extracted from cells using FreeZol Reagent according to the manufacturer’s instructions (Vazyme, Nanjing, China). Reverse transcription

(RT) was performed with HiScript Q RT SuperMix (Vazyme, Nanjing, China) for qPCR. RT–PCR was performed in triplicate with a SYBR Green PCR Kit (Vazyme, Nanjing, China) on QuantStudio™ 5 Real-Time Fluorescent Quantitative PCR System (Applied Biosystems). The relative expression levels of the target genes were calculated using the comparative $2^{-\Delta\Delta Ct}$ method. The primers used are listed in Supplementary Table 3.

RNA Sequencing (RNA-seq)

Total RNA was first extracted from WT and Nat10-KO MC38 cells for RNA-seq, and the quality and quantity of the RNA were assessed with a NanoDropTM ND-1000 spectrophotometer. Denaturing agarose gel electrophoresis was used to assess RNA integrity. mRNA extraction was performed using the a NEBNextR Poly(A) mRNA Magnetic Isolation Module. The RNA libraries were constructed using a KAPA Stranded RNA-Seq Library Prep Kit (Illumina). Libraries were sequenced using the Illumina HiSeq 4000 platform. RNA-seq was completed by Gene Denovo (Guangzhou, China).

Unique Identifier RNA Sequencing (UID RNA-seq)

UID RNA-seq experiments and high through-put sequencing and data analysis were conducted by SeqHealth Technology Co., Ltd. (Wuhan, China). Total RNA was extracted from $CD8^{+}$ T cells treated with or without rDKK2 using TRIzol Reagent (Invitrogen, cat. NO 15596026. Two micrograms of total qualified RNA

was used for stranded RNA sequencing library preparation using KC-Digital™ Stranded mRNA Library Prep Kit for Illumina® (catalog NO. DR08502, Wuhan Seqhealth Co., Ltd. China) following the manufacturer's instructions. The library products corresponding to 200-500 bps were enriched, quantified and finally sequenced on DNBSEQ-T7 sequencer (MGI Tech Co., Ltd. China) with the PE150 configuration.

Single-cell RNA sequencing (scRNA-seq) and data analysis

scRNA-seq was performed by Novel Bioinformatics Co., Ltd. (Shanghai, China). Intestinal tumor samples collected from *Nat10*$^{f/f}$ or *Nat10*^{cKO} mice were used for scRNA-seq analysis. Briefly, fresh mucosal samples were dissected using iris scissors and digested in a PBS-collagen II/IV solution for 30 min at 37 °C with agitation at 800 rpm. Following digestion, a 45-μm filter was used to filter the cell suspension. Centrifugation at 500 rpm for 6 min at 4 °C was performed to remove dead cells and red blood cells. The cells were then washed, resuspended in PBS supplemented with 0.5% FBS, and loaded into microfluidic channels. A droplet-based sequencing platform (10x Genomics) was used to generate a cDNA library. Library preparation was performed with the Single Cell 3′ Library Gel Bead Kit V2 (10x Genomics), and the library was subsequently sequenced on an Illumina NovaSeq 6000 system, aiming for a minimum of 100,000 150-bp paired-end reads per cell.

ac4C-modified RNA immunoprecipitation sequencing (acRIP-seq)

To quantify the ac4C modification levels of specific genes, purified total RNA exceeding 150 μg was obtained, and the integrity and quantity of each RNA sample were assessed via agarose gel electrophoresis and a NanoDrop™ spectrophotometer. In accordance with the manufacturer's protocol, intact mRNA was first isolated from the total RNA samples using an Arraystar Seq Star™ poly(A) mRNA isolation kit. The isolated mRNA was then chemically fragmented into 100-nucleotide-long fragments via incubation in lysis buffer (10 mM Zn^{2+} and 10 mM Tris-HCl, pH 7.0), and the size of the fragmented mRNA was confirmed by agarose gel electrophoresis. Subsequently, ac4C-modified mRNAs were immunoprecipitated using anti-ac4C antibodies (with a portion of the fragmented mRNAs retained as input). The main downstream procedures included immunoprecipitation, washing, and elution. The eluted ac4C mRNA fragments were then concentrated for RNA-seq library preparation. RNA-seq libraries for both ac4C antibody-enriched mRNAs and input mRNAs were prepared using the KAPA Stranded mRNA-seq Kit (Illumina). The prepared libraries were diluted to a final concentration of 8 pM, and clusters were generated on an Illumina-cBot system using the HiSeq 3000/4000 PE Cluster Kit (#PE-410-1001, Illumina) before sequencing on the Illumina HiSeq 4000 platform. For acRIP-seq data analysis, raw reads were trimmed using Trimmatic software and aligned to the Ensembl reference genome using HISAT2 software (v2.1.0). Differentially enriched regions (peaks) identified by acRIP-seq

between groups were analyzed using exomePeak software. These differential peaks were annotated using the latest Ensembl database. Sequence motifs, which constitute one of the fundamental functional units of molecular evolution, were identified within ac4C peak sequences using the Multiple Em for Motif Elicitation (MEME) and Discriminative Regular Expression Motif Elicitation (DREME) algorithms. acRIP-seq was provided by Guangzhou Epibiotek Co., Ltd. (Guangzhou, China).

NaCNBH3-based chemical ac4C sequencing (ac4C-seq) and data analysis

RNA extraction, bisulfite treatment, library preparation and high through-put sequencing were conducted by SeqHealth Technology Co., Ltd. (Wuhan, China). Total RNA was extracted from WT and *Nat10*-KO MC38 cells using TRIzol Reagent (Invitrogen, cat. NO 15596026). To be specific, 30 μg of total RNA was used for rRNA depletion through Ribo-off rRNA Depletion Kit (Catalog NO. N409-02, vazyme). Then for each conversion reaction, RNA (1 μg) was incubated with either $NaCNBH_3$ (100 mM in H_2O + 100 mM HCl) (denoted as "BH") or untreated 'mock' control (H_2O + 100 mM HCl) (denoted as "Con"). In a final reaction volume of 100 μL, samples were incubated for 20 minutes at 20 ℃. Reactions were stopped by neutralization of pH by the addition of 30 μL 1 M Tris-HCl pH 8.0. Reactions were adjusted to 200 μL with H_2O, precipitated with ethanol, desalted with 70% ice-cold ethanol, briefly dried on Speedvac, resuspended in H_2O. Both the "Con" and "BH" RNA were used as input for

RNA sequencing library preparation using a KC^{TM} Digital Stranded Total RNA-seq Library Prep Kit for Illumina® (catalog NO. DR087-02, Wuhan SeqHealth Co., Ltd. China) following the manufacturer's instructions. PCR products corresponding to 200-500 bps were enriched, quantified and finally sequenced on NovaSeq 6000 sequencer (Illumina) with the PE150 configuration.

References

1. Wang Q, Chen C, Ding Q, Zhao Y, Wang Z, Chen J, et al. METTL3-mediated m(6)A modification of HDGF mRNA promotes gastric cancer progression and has prognostic significance. *Gut.* 2020;69(7):1193-205.
2. Wang Z, Wang Q, Chen C, Zhao X, Wang H, Xu L, et al. NNMT enriches for AQP5(+) cancer stem cells to drive malignant progression in early gastric cardia adenocarcinoma. *Gut.* 2023;73(1):63-77.
3. Weichert W, Roske A, Gekeler V, Beckers T, Ebert MP, Pross M, et al. Association of patterns of class I histone deacetylase expression with patient prognosis in gastric cancer: a retrospective analysis. *Lancet Oncol.* 2008;9(2):139-48.
4. Wang S, Wu X, Chen Y, Zhang J, Ding J, Zhou Y, et al. Prognostic and predictive role of JWA and XRCC1 expressions in gastric cancer. *Clin Cancer Res.* 2012;18(10):2987-96.
5. Gui J, Zahedi F, Ortiz A, Cho C, Katlinski KV, Alicea-Torres K, et al. Activation of p38alpha stress-activated protein kinase drives the formation of the pre-metastatic niche in the lungs. *Nat Cancer.* 2020;1(6):603-19.
6. Hu C, Qiao W, Li X, Ning ZK, Liu J, Dalangood S, et al. Tumor-secreted FGF21 acts as an immune suppressor by rewiring cholesterol metabolism of CD8(+)T cells. *Cell Metab.* 2024;36(3):630-47 e8.
7. Zhang W, Liu Y, Yan Z, Yang H, Sun W, Yao Y, et al. IL-6 promotes PD-

L1 expression in monocytes and macrophages by decreasing protein tyrosine phosphatase receptor type O expression in human hepatocellular carcinoma. *J Immunother Cancer.* 2020;8(1).

8. Moreno Valtierra M, Urue Corral A, Jimenez-Avalos JA, Barbosa Avalos E, Davila-Rodriguez J, Morales Hernandez N, et al. Patterned PVA Hydrogels with 3D Petri Dish((R)) Micro-Molds of Varying Topography for Spheroid Formation of HeLa Cancer Cells: In Vitro Assessment. *Gels.* 2024;10(8).

9. Chen C, Shen N, Chen Y, Jiang P, Sun W, Wang Q, et al. LncCCLM inhibits lymphatic metastasis of cervical cancer by promoting STAU1-mediated IGF-1 mRNA degradation. *Cancer Lett.* 2021;518:169-79.

10. Wang Q, Li M, Chen C, Xu L, Fu Y, Xu J, et al. Glucose homeostasis controls N-acetyltransferase 10-mediated ac4C modification of HK2 to drive gastric tumorigenesis. *Theranostics.* 2025;15(6):2428-50.

Supplementary figure legends

Supplementary Figure S1. *NAT10* depletion attenuates tumor progression and activates anti-tumor immunity in CRC allografts. (**A**) Schematic for establishing allografts derived from MC38 WT and *Nat10*-KO cells in BALB/c-nude mice. (**B** and **C**) Tumor growth curves (mean ± SEM) (**B**), representative images of tumors from each group (left panel) and tumor weights (right panel) (**C**) (n = 9 mice/group). (**D** and **E**) Representative mIHC staining of cytotoxic Perforin$^+$ and exhausted LAG3$^+$ CD8$^+$ T cell proportions in tumor sections (n = 3 mice/group). (**F**) Western blot confirming *Nat10* knockout in CT-26 cells. (**G**) Schematic for establishing allografts from derived CT-26 WT or *Nat10*-KO cells in BALB/c mice. (**H** and **I**) Tumor growth curves (mean ± SEM) (**H**), representative images of tumor-bearing mice from each group (left panel) and tumor weights (right panel) (**I**) (n = 6 mice/group). (**J**) Flow cytometry analysis of the composition of immune cells in tumors from the CT-26 WT and *Nat10*-KO groups (n = 4 mice/group). (**K** and **L**) Flow cytometry analysis of GzmB$^+$ and IFN-γ$^+$ CD8$^+$ T cells infiltration in the CT-26 WT and *Nat10*-KO tumors (n = 4 mice/group). (**M**) Flow cytometry analysis of Tebt$^+$ and IFN-γ$^+$ CD4$^+$ T cell populations in CT-26 WT and *Nat10*-KO tumors (n = 4 mice/group). All data are shown as the mean ± SD of indicated mice per group. Statistical analysis was performed by Two-way ANOVA (B, H), two-tailed Student's t-test (C, J, L, M) or One-way ANOVA (D, E, I). ns, $p \geq 0.05$, not significant; $p < 0.05$, statistically significant.

Supplementary Figure S2. Intestine epithelial *Nat10* deficiency suppresses colorectal tumorigenesis. (**A**) Schematic of the CRISPR-Cas9-targeted construct for generating *Nat10*^{cKO} mice. (**B**) Genotyping validation of PCR-amplified genomic DNA from wild-type (WT), heterozygous (HET), and conditional knockout (cKO) mice via agarose gel electrophoresis. Loxp-floxed *Nat10* Prime 1: WT band, 186 bp; Targeted band, 290 bp; Loxp-floxed *Nat10* Prime 2: WT band, 0 bp; Targeted band, 337 bp; Villin1-Cre prime: Control band, 324 bp; Transgene band, 195 bp. (**C**) Representative H&E staining and Ki67 IHC staining of CRC tumors from AOM/DSS-induced *Nat10*$^{fl/fl}$ and *Nat10*^{cKO} mice (n = 5 mice/group). (**D**) UMAP plot of single-cell transcriptomic profiles, illustrating distinct cellular compositions in tumors from *Nat10*$^{fl/fl}$ mice compared with *Nat10*^{cKO} mice.

Supplementary Figure S3. NAT10 expression exhibits a negative correlation with CD8^{+} T cell immune responses. (**A**) ESTIMATE algorithm analysis of the correlation between NAT10 expression and the immune score in a CRC cohort (GSE131418 dataset) (n = 544). (**B**) Analysis of differential immune cell infiltration in the TME of CRC patients with high/low *NAT10* expression based on the TCGA database (https://tcga-data.nci.nih.gov/tcga/). (**C** and **D**) ESTIMATE algorithm analysis of the correlation between *NAT10* expression and the proportion of activated CD8^{+} T cells or effector memory CD8^{+} T cells in CRC TME based on the TCGA database (https://tcga-

data.nci.nih.gov/tcga/). (**E**) CellChat algorithm analysis of ligand-receptor-mediated interactions between tumor cells and TME cells in CRC tumors from *Nat10*$^{f/f}$ and *Nat10*^{cKO} mice based on scRNA-seq data. (**F**) Differential intercellular communication networks in CRC tumors from *Nat10*^{cKO} versus *Nat10*$^{f/f}$ mice, highlighting epithelial-derived ligands (senders) and TME cell cognate receptors (receptors). The line thickness reflects the interaction strength. (**G**) Altered cellular communication patterns between the CRC tumors of *Nat10*^{cKO} and *Nat10*$^{f/f}$ mice.

Supplementary Figure S4. Intestine epithelial *Nat10* deficiency enhances CD8$^+$ T cell-mediated antitumor immunity. (**A**) Experimental design for CD4$^+$/CD8$^+$ T cell depletion in MC38 WT or *Nat10*-KO allograft-bearing mice. (**B**) Representative flow cytometry analysis of CD4$^+$ and CD8$^+$ T cells depletion efficiency in the spleens of treated mice (n = 7 mice/group). (**C**-**E**) Tumor growth curves (**C**) (mean ± SEM), representative tumors image (**D**), and tumor weights (**E**) (n = 7 mice/group). (**F**) Experimental design for macrophage depletion in MC38 WT or *Nat10*-KO allograft-bearing mice. (**G**) Representative flow cytometry analysis of macrophage depletion efficiency in the spleens of treated mice (n = 6 mice/group). (**H**-**J**) Tumor growth curves (mean ± SEM) (**H**), representative tumor images (**I**), and tumor weights (**J**) (n = 6 mice/group). Data are shown as the mean ± SD of indicated mice per group (C, E, H, J). All

statistical analysis was performed by Two-way ANOVA. ns, $p \geq 0.05$, not significant; $p < 0.05$, statistically significant.

Supplementary Figure S5. Tumor-intrinsic NAT10 deficiency enhances CD8$^+$ T cell infiltration and cytotoxic functions. (**A**) Flow cytometry of CD8$^+$ T cell migration toward CM from CT-26 WT/*Nat10*-KO cells. (**B**) Western blot of MC38 cells overexpressing *Nat10*. (**C**) Flow cytometry of CD8$^+$ T cell migration toward CM from MC38 WT/*Nat10*-OE cells. (**D** and **E**) OT-1 CD8$^+$ T cells co-cultured with CT-26-OVA WT/*Nat10*-KO cells (1:1, 24h). Flow cytometry of GzmB$^+$/IFN-γ$^+$ CD8$^+$ T cells. (**F**) Western blot of *Nat10* knockout in B16F10-OVA cells. (**G** and **H**) OT-1 CD8$^+$ T cells co-cultured with OVA-expressing B16F10 WT/*Nat10*-KO cells (1:1, 24h). Flow cytometry of GzmB$^+$/IFN-γ$^+$ CD8$^+$ T cells. (**I**) OT-1 CD8$^+$ T cells co-cultured with OVA-expressing CT-26/B16F10 WT/*Nat10*-KO cells. LDHA release assays for OT-1 CD8$^+$ T cell cytotoxicity. (**J**-**K**) OT-1 CD8$^+$ T cells co-cultured with OVA-expressing CT-26 WT/ *Nat10*-KO cells in a 3D-culture system. Representative images of tumor spheroid disintegration (**J**) and mIHC staining (apoptotic tumor cells: cleaved Caspase-3, red; CD8$^+$ T cell infiltration, green) (**K**). (**L**) Representative H&E/IHC staining (CDX2, Ki67, NAT10) in human CRC organoids (n = 3). (**M**) Western blot of *NAT10* knockout in human CRC organoids. (**N**) Human CRC organoids co-cultured with autologous peripheral blood-derived CD8$^+$ T cells. Flow cytometry of apoptosis in WT/NAT10-KO organoids (n = 3). The data are presented as the

mean ± SD of three independent experiments (A, C, E-I, N). One of three representative experiments is shown (J, K, L). Statistical analysis was performed by One-way ANOVA (A, E, H, I) or two-tailed Student's t-test (C, N). ns, $p \geq 0.05$, not significant; $p<0.05$, statistically significant.

Supplementary Figure S6. NAT10 directly regulates *DKK2* mRNA stability via ac4C modification. (**A**) A representative pie chart depicting the proportions of total ac4C peaks across mRNA regions, including the 3'-untranslated region (3'-UTR), 5'-untranslated region (5'-UTR), coding sequence (CDS), transcription start site (TSS), start codon and stop codon. (**B**) Histogram of C-to-T conversion at the ac4C site of *Dkk2* mRNA in both WT and *Nat10*-KO cells with or without sodium cyanoborohydride (NaCNBH3) treatment. The black triangle indicates the ac4C site, with the surrounding transcript sequence displayed. For the ac4C site, the blue bars represent cytosine, the red bars represent thymine, and the gray bars indicate other positions. The C-to-T mutation rate and track heights are shown on the right side of each panel. (**C**) qRT–PCR analysis of *Dkk2* mRNA levels in CT-26 WT and *Nat10*-KO cells. (**D**) qRT–PCR analysis of *Dkk2* mRNA levels in CT-26 WT and *Nat10*-KO cells treated with actinomycin D (2.5 μg/mL) at the indicated time points. The data are presented as the mean ± SD of three independent experiments (C, D). Statistical analysis was performed by One-way ANOVA (C) or Two-way ANOVA (D). ns, $p \geq 0.05$, not significant; $p<0.05$, statistically significant.

Supplementary Figure S7. NAT10 modulates CD8$^+$ T cell recruitment and cytotoxicity through DKK2 regulation. (**A**) Western blot analysis of Dkk2 protein expression in B16F10 *Nat10*-KO cells. (**B**) Western blot analysis of DKK2 protein expression in *NAT10*-KO (HCT116) and *NAT10*-OE (SW620) human CRC cells. (**C** and **D**) ELISA quantification of secreted DKK2 in conditioned media from *NAT10*-KO and OE cells. (**E**) Western blot analysis of Dkk2 levels in AOM/DSS-induced tumors from *Nat10*$^{f/f}$ and *Nat10*^{cKO} mice (n = 5 mice/group). (**F**-**K**) OT-1 CD8$^+$ T cells were co-cultured with OVA-expressing CT-26/B16F10 WT and *Nat10*-KO cells at a ratio of 1:1 with or without rDkk2 treatment for 24 h. Flow cytometry analysis of the GzmB$^+$ and IFN-γ$^+$ CD8$^+$ T cell populations. (**L**) LDHA release assay evaluates cytotoxic capacity of OT-1 CD8$^+$ T cells. The data are presented as the mean ± SD of three independent experiments (C, D, H, K, L). Statistical analysis was performed by One-way ANOVA (C, left panel of D) or two-tailed Student's t-test (right panel of D, H, K, L). ns, $p \geq 0.05$, not significant; $p < 0.05$, statistically significant.

Supplementary Figure S8. DKK2 promotes cholesterol biosynthesis to suppress CD8$^+$ T cell anti-tumor function. (**A** and **B**) Flow cytometry analysis of the population of GzmB$^+$ and IFN-γ$^+$ CD8$^+$ T cells after 24 h of culture with rDkk2 following pretreatment with SOST. (**C**) KEGG analysis of significantly

enriched pathways of $CD8^+$ T cells treated with or without recombinant DKK2 (rDkk2). (**D**) scRNA-seq analysis of the enrichment of gene signature scores in tumor-infiltrating effector $CD8^+$ T cells from *Nat10*$^{fl/fl}$ and *Nat10*^{cKO} mice. (**E** and **F**) Cholesterol levels in activated $CD8^+$ T cells cultured with CT-26/B16F10 WT/Nat10-KO conditioned media (CM) for 24 hours. (**G** and **H**) Flow cytometry $GzmB^+$ and IFN-γ^+ $CD8^+$ T cell with cholesterol-supplemented CT-26/B16F10 WT or *Nat10*-KO cells CM for 24 h. (**I**) Flow cytometry of p-AKT and p-mTOR levels in activated $CD8^+$ T cells cultured with B16F10 WT or *Nat10*-KO cells CM for 24 h. The data are presented as the mean ± SD of three independent experiments (B, E-I). Statistical analysis was performed by Two-way ANOVA (B), One-way ANOVA (E, F, I) or two-tailed Student's t-test (G, H). ns, $p \geq 0.05$, not significant; $p < 0.05$, statistically significant.

Supplementary Figure S9. NAT10/DKK2 axis promotes cholesterol accumulation in $CD8^+$ T cells impair their cytotoxicity. (**A**) Western blot analysis of p-mTOR/mTOR levels in $CD8^+$ T cells after rapamycin treatment. (**B**) Representative IHC staining of Ki67 and cleaved Caspase-3 in frozen sections of tumor tissues (n = 5 mice/group). (**C**) Representative Filipin III staining for cholesterol and mIHC staining for $CD4^+$ T cells, $CD8^+$ T cells, and macrophages in frozen sections of tumor tissues (n = 3 mice/group). (**D**) Western blot analysis was conducted to evaluate the efficiency of *Lrp5* and *Lrp6* knockdown in $CD8^+$ T cells 48 h post-infection with respective shRNA lentiviruses.

Supplementary Figure S10. Dual targeting of NAT10 or DKK2 synergizes with PD1 blockade to suppress CRC. (**A**) Schematic of the combined treatment with anti-PD-1 antibody and Remodelin. (**B** and **C**) Representative tumor images (n = 7 mice/group) and IHC staining of Ki67 and cleaved Caspase-3 in tumor tissues (n = 5 mice/group). (**D**) Schematic of the combined treatment with anti-PD-1 and anti-DKK2 antibody (5F8). (**E** and **F**) Representative tumor images (n = 7 mice/group) and IHC staining of Ki67 and cleaved Caspase-3 in tumor tissues (n = 5 mice/group).

Supplementary Figure S11. Receiver operating characteristic (ROC) curves were obtained to show the relation between area under the curve (AUC) at different cutoff values of NAT10 (A) or DKK2 (B) immunoreactivity score (IRS) for 5 years of overall survival time.

Supplementary Figure S12. Gating strategies. (**A**) Sequential gating strategy of tumor-infiltrating immune cells from tumor tissue for flow cytometry analysis (Figure 1F, 2H, 8E, 9B and 9E, Supplementary Figure 1J). (**B**) Sequential gating strategy of $CD4^+$ and $CD8^+$ T cell related phenotypes from tumor tissue for flow cytometry analysis (Figure 1I, 1L, 2I, 8F, 9C and 9F, Supplementary Figure 1L, 1M). (**C**) Sequential gating strategies of p-AKT and p-mTOR levels in tumor-

infiltrating $CD8^+$ T cells for flow cytometry analysis (Figure 8H). (**D**) Sequential gating strategies of cholesterol level in tumor-infiltrating $CD8^+$ T cells for flow cytometry analysis (Figure 8G). The respective gate names are given in the corresponding figures.
