# Supplementary material for: Targeting the N-acetyltransferase 10/DKK2 axis enhances CD8+ T cell antitumor activity in colorectal cancer models
Source: J Clin Invest. 2026 Jan 16;136(2):e196722. doi: 10.1172/JCI196722 (PMC12807479; doi:10.1172/JCI196722)

Full unedited blot for Figure 1A

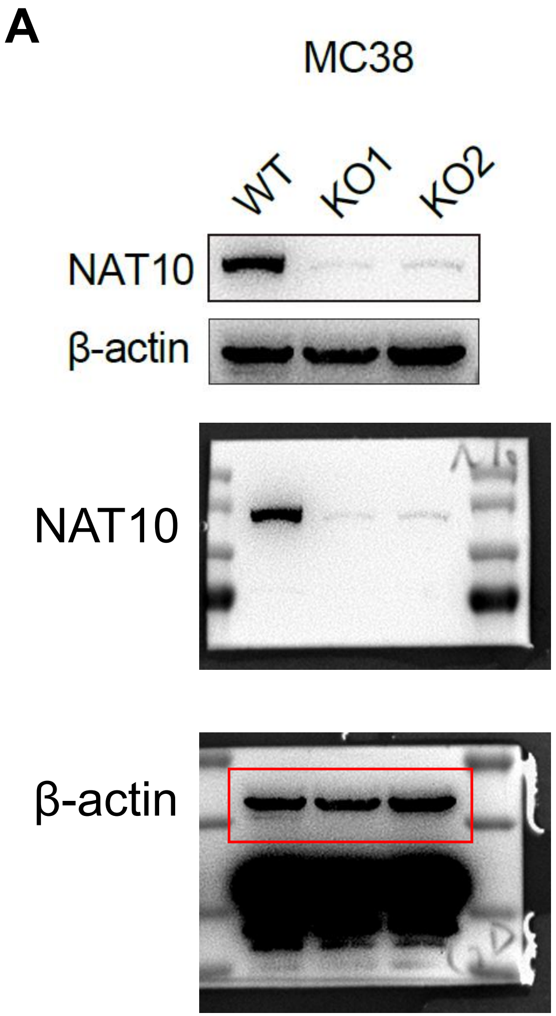

Full unedited blot for Figure 5A

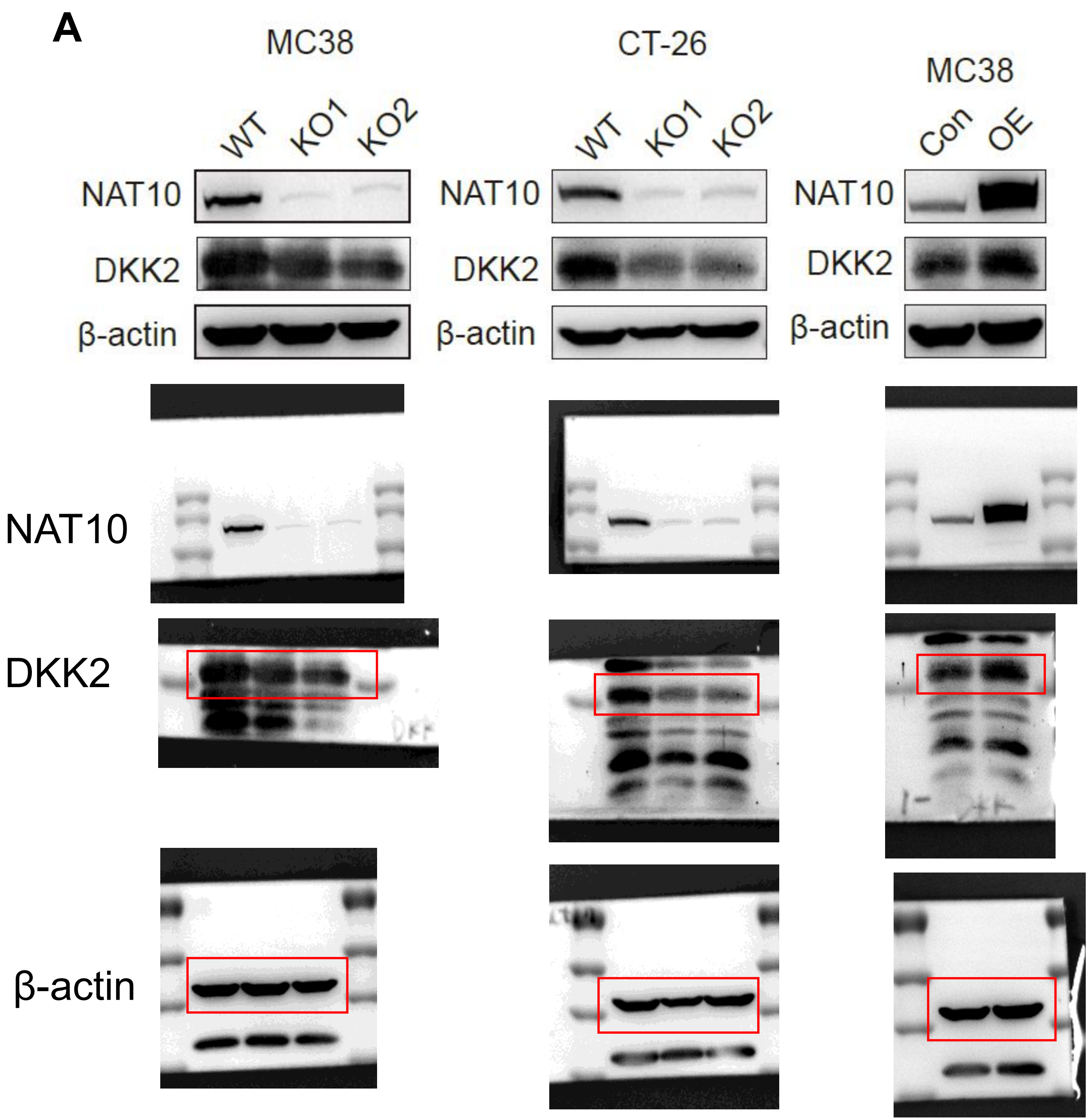

Full unedited blot for Figure 7E

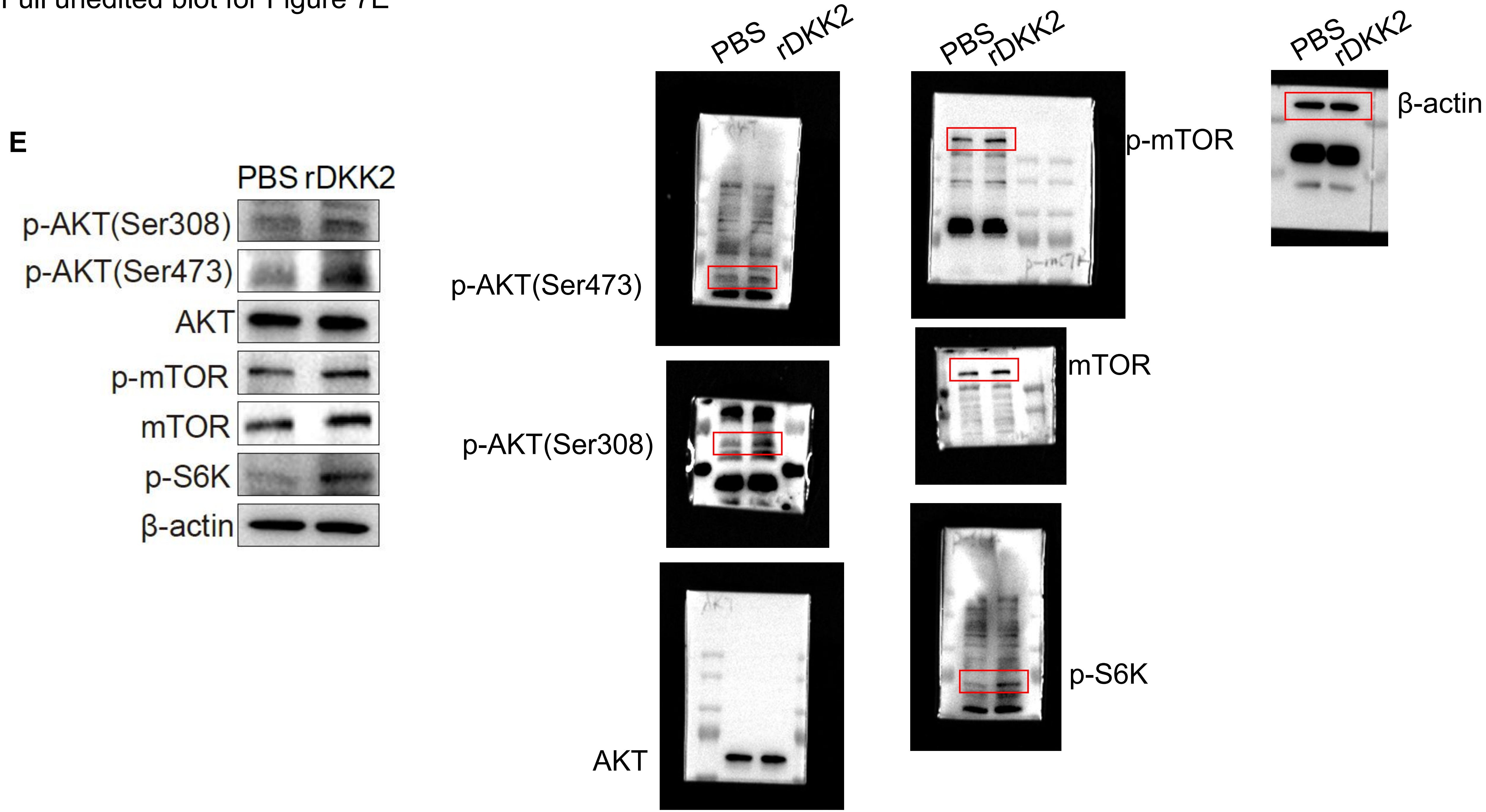

Full unedited blot for Figure 8J

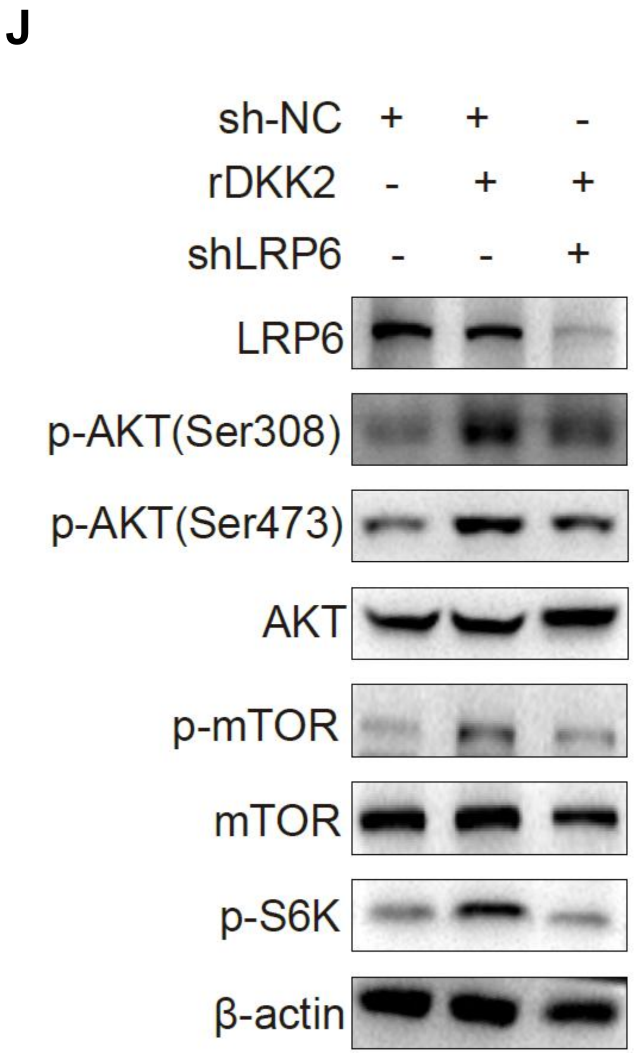

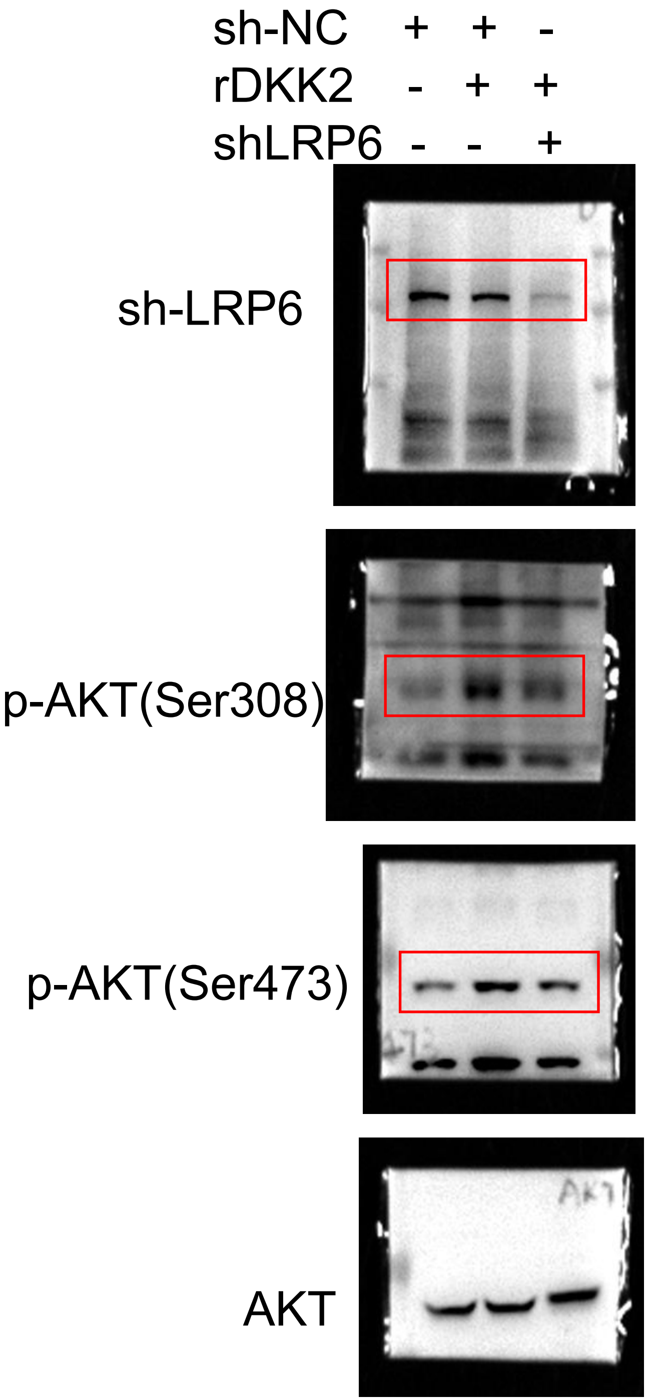

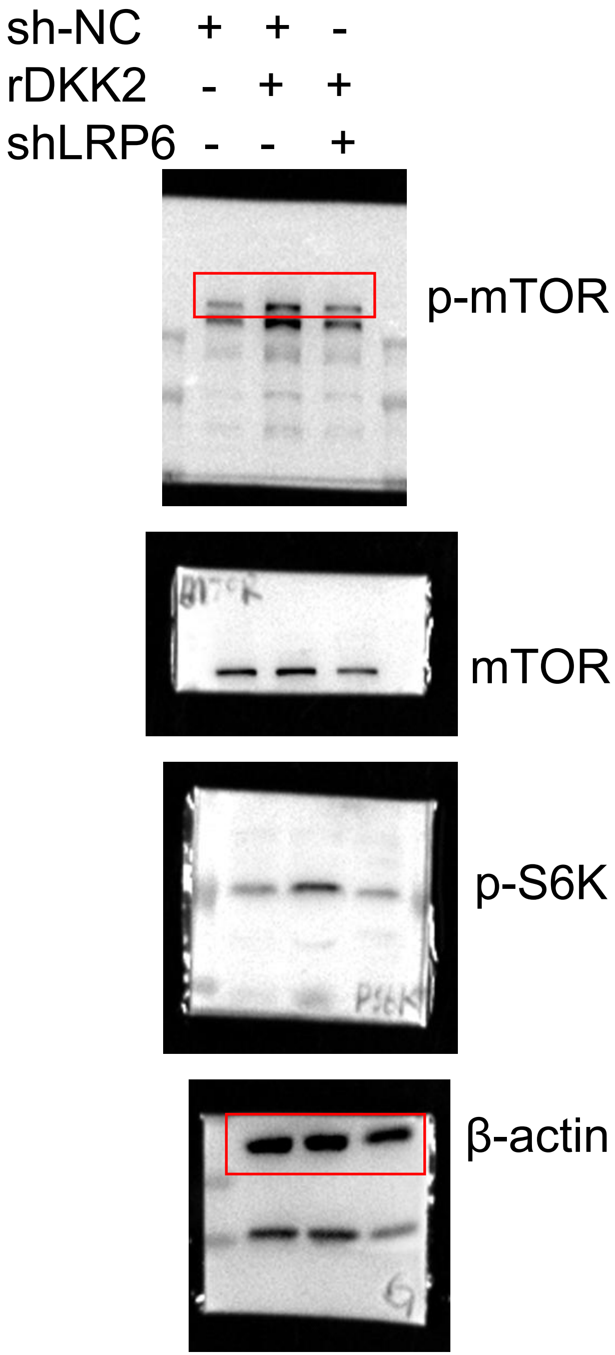

Full unedited blot for Supplementary Figure 1F

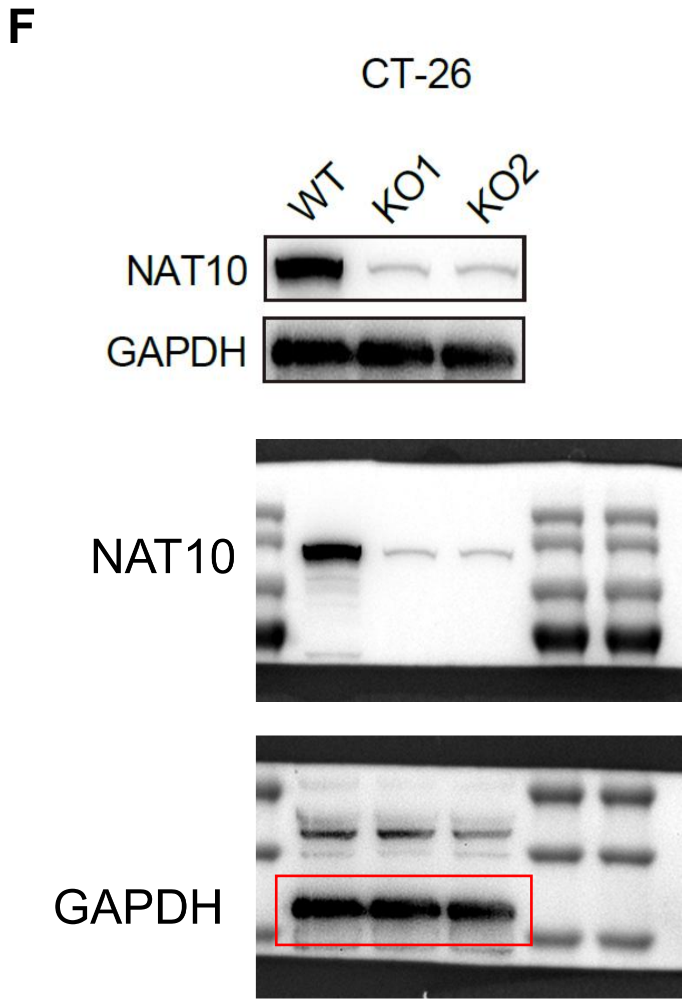

Full unedited blot for Supplementary Figure 5B

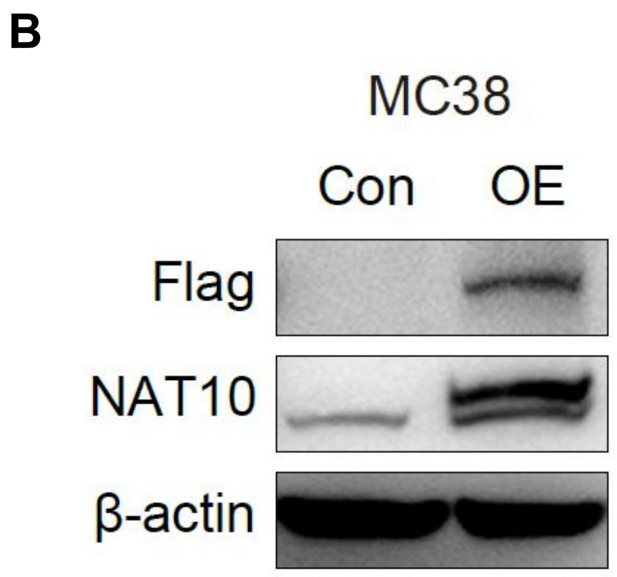

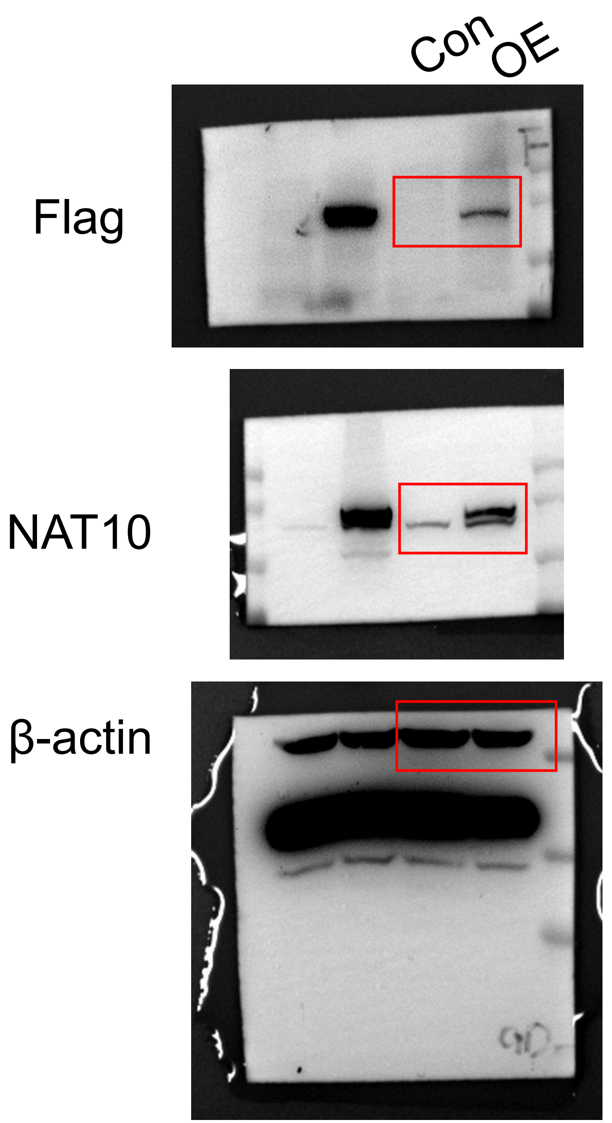

Full unedited blot for Supplementary Figure 5F

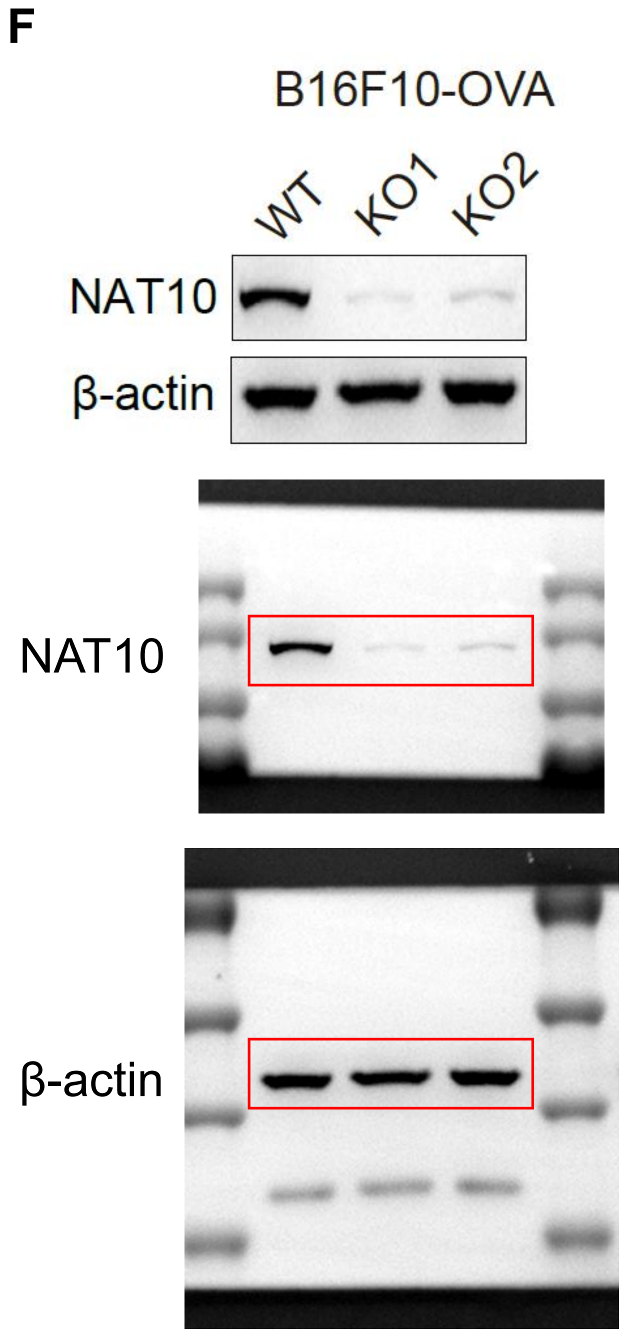

Full unedited blot for Supplementary Figure 5M

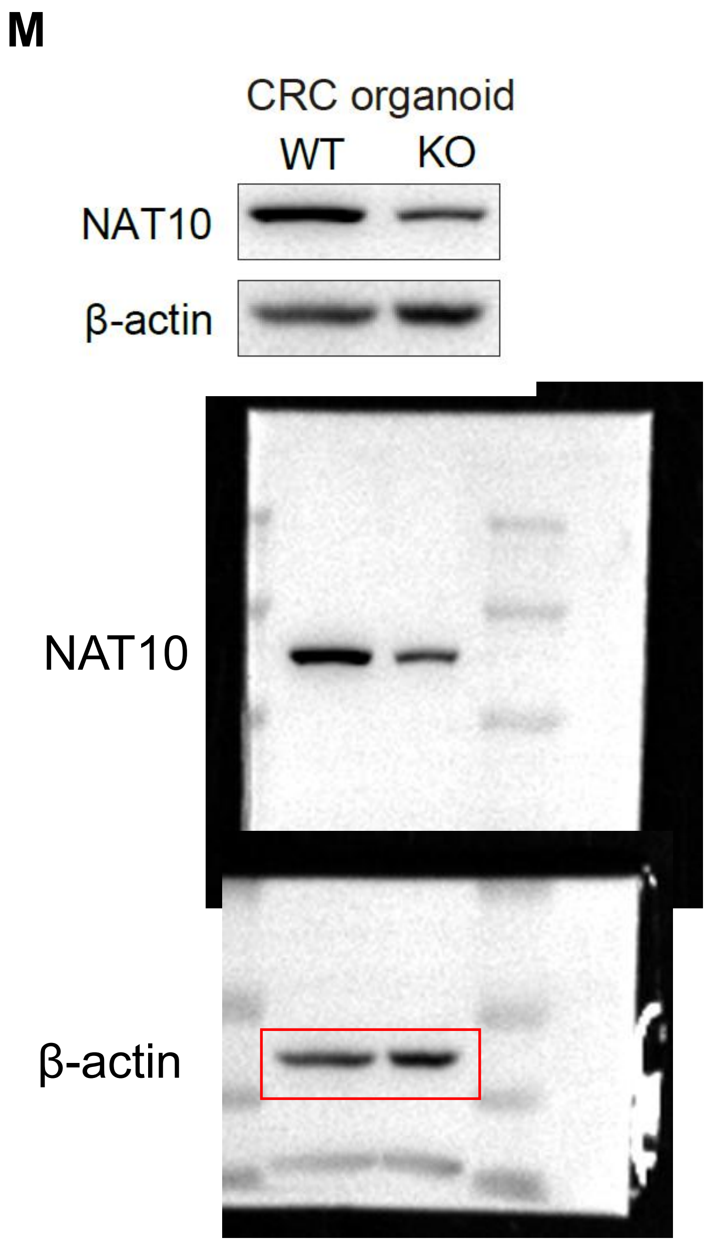

Full unedited blot for Supplementary Figure 7A

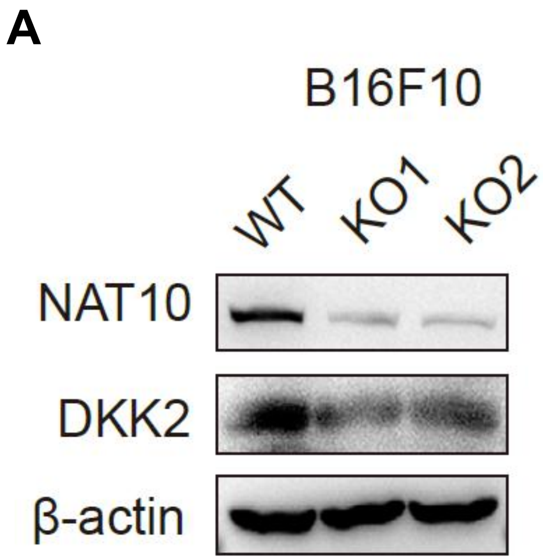

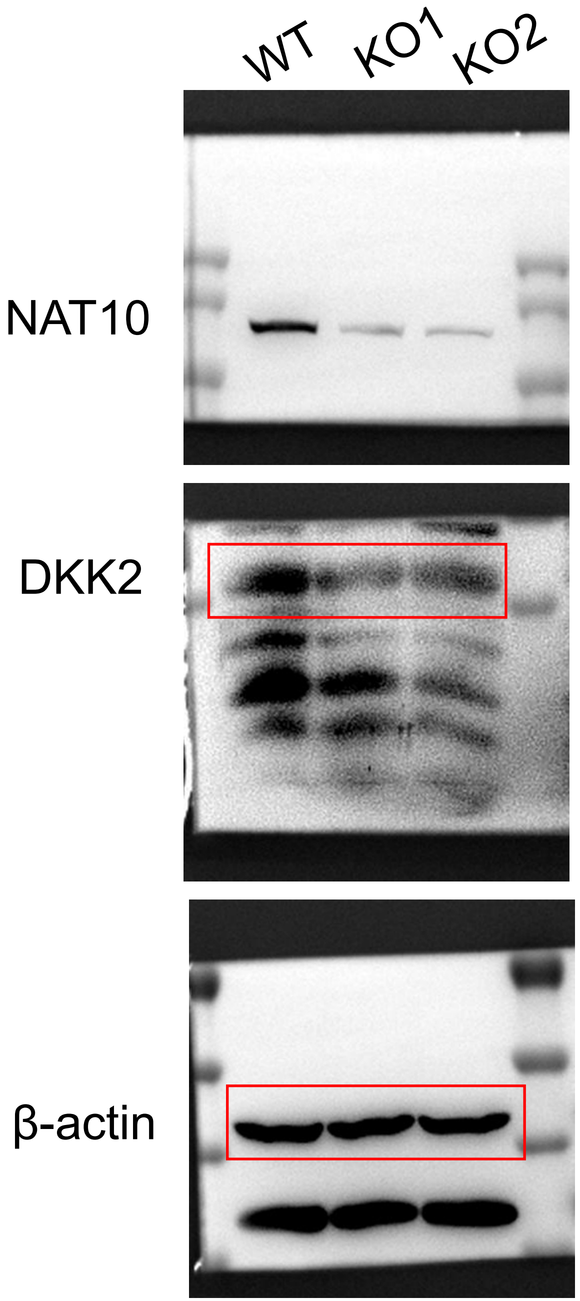

Full unedited blot for Supplementary Figure 7B

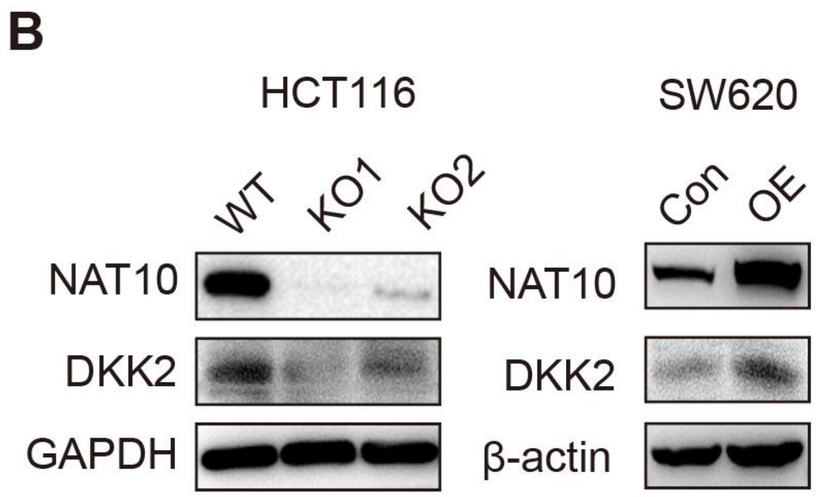

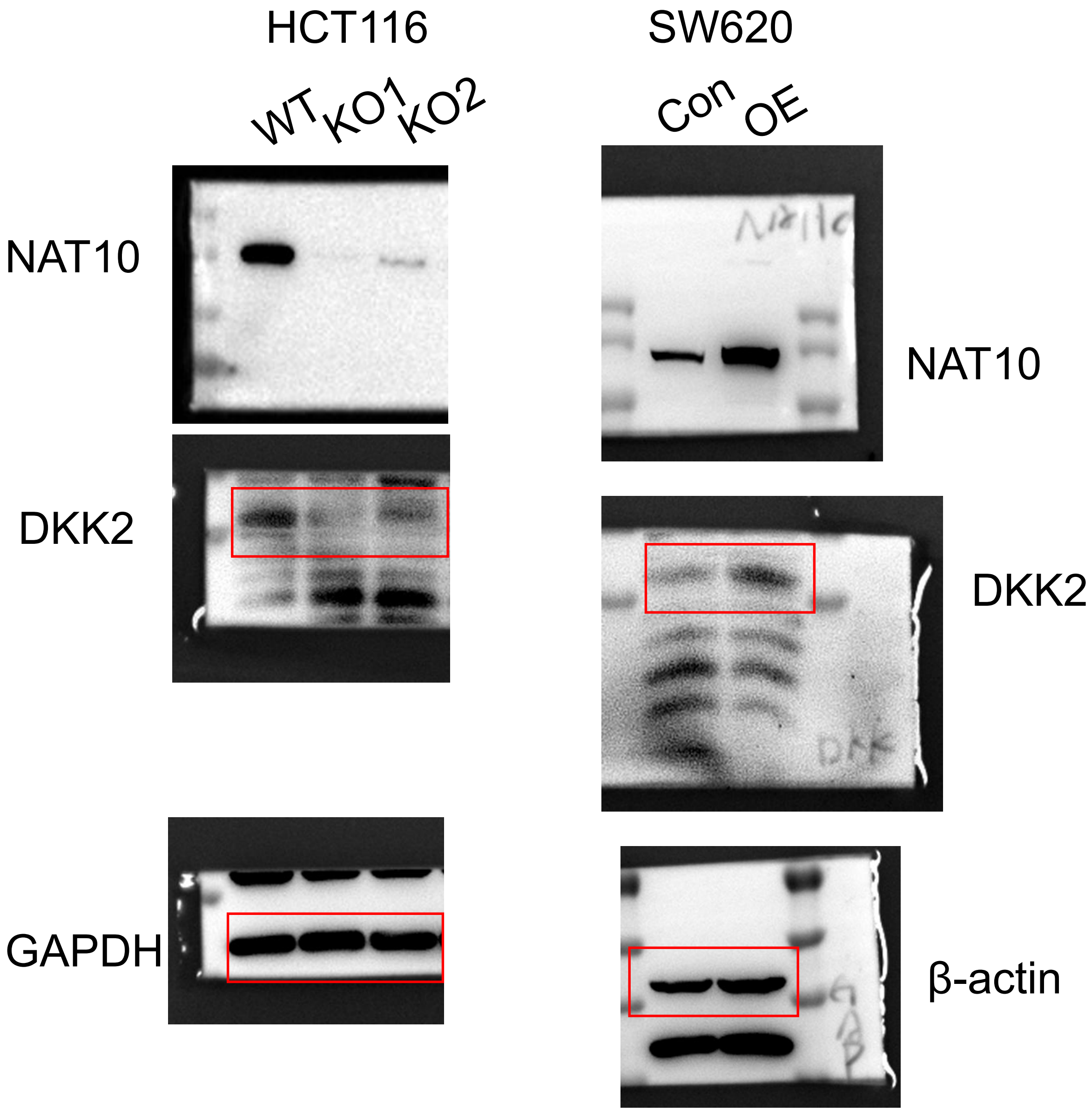

Full unedited blot for Supplementary Figure 7E

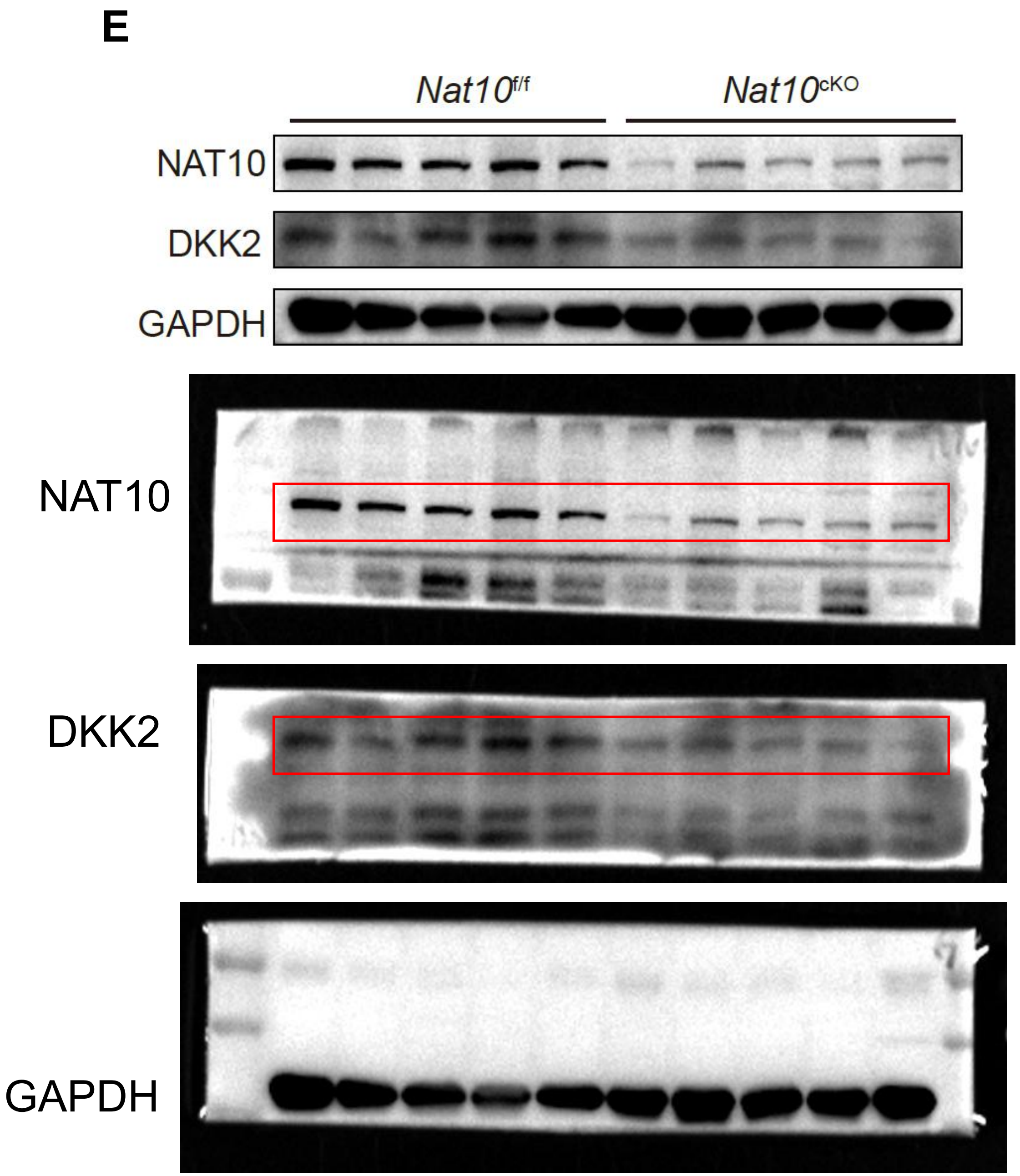

Full unedited blot for Supplementary Figure 9A

A

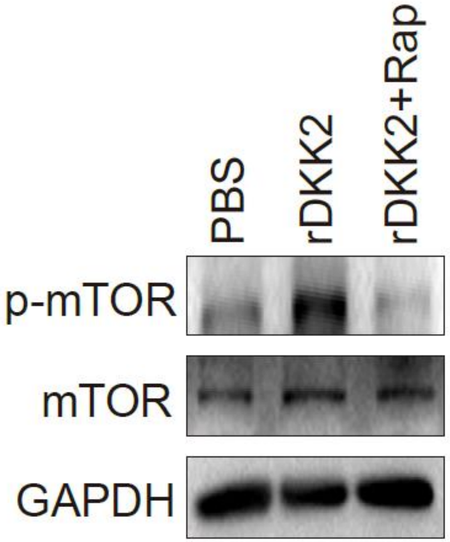

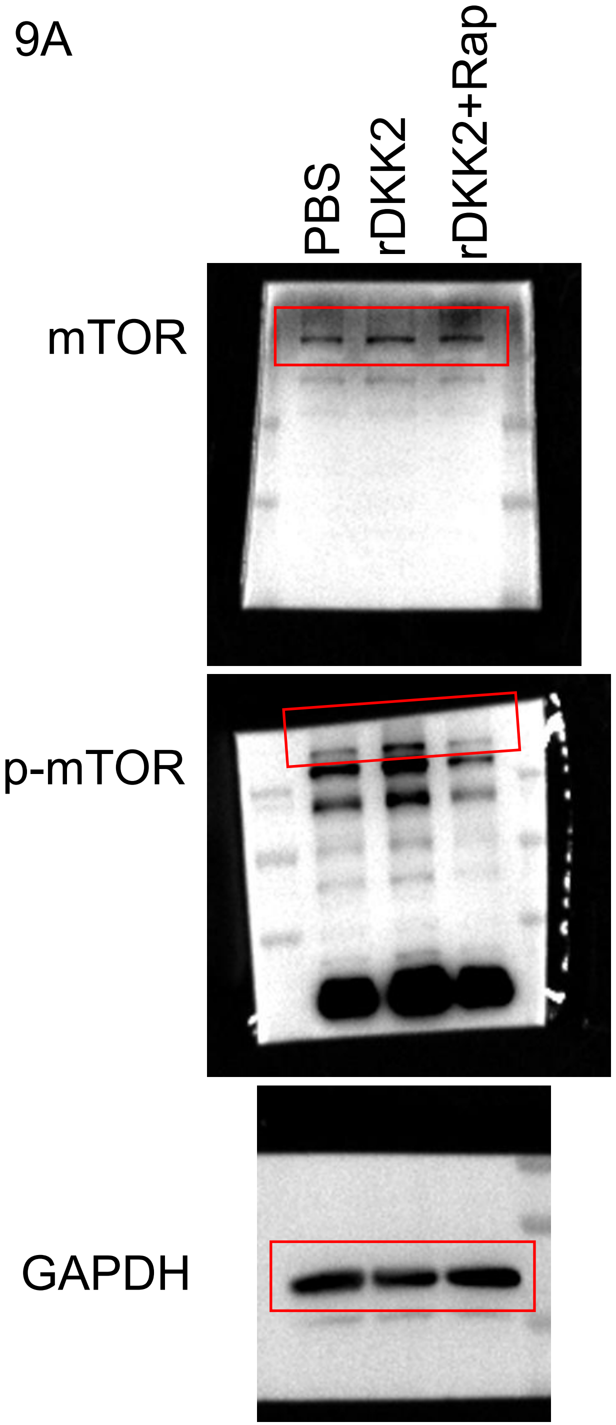

Full unedited blot for Supplementary Figure 9D

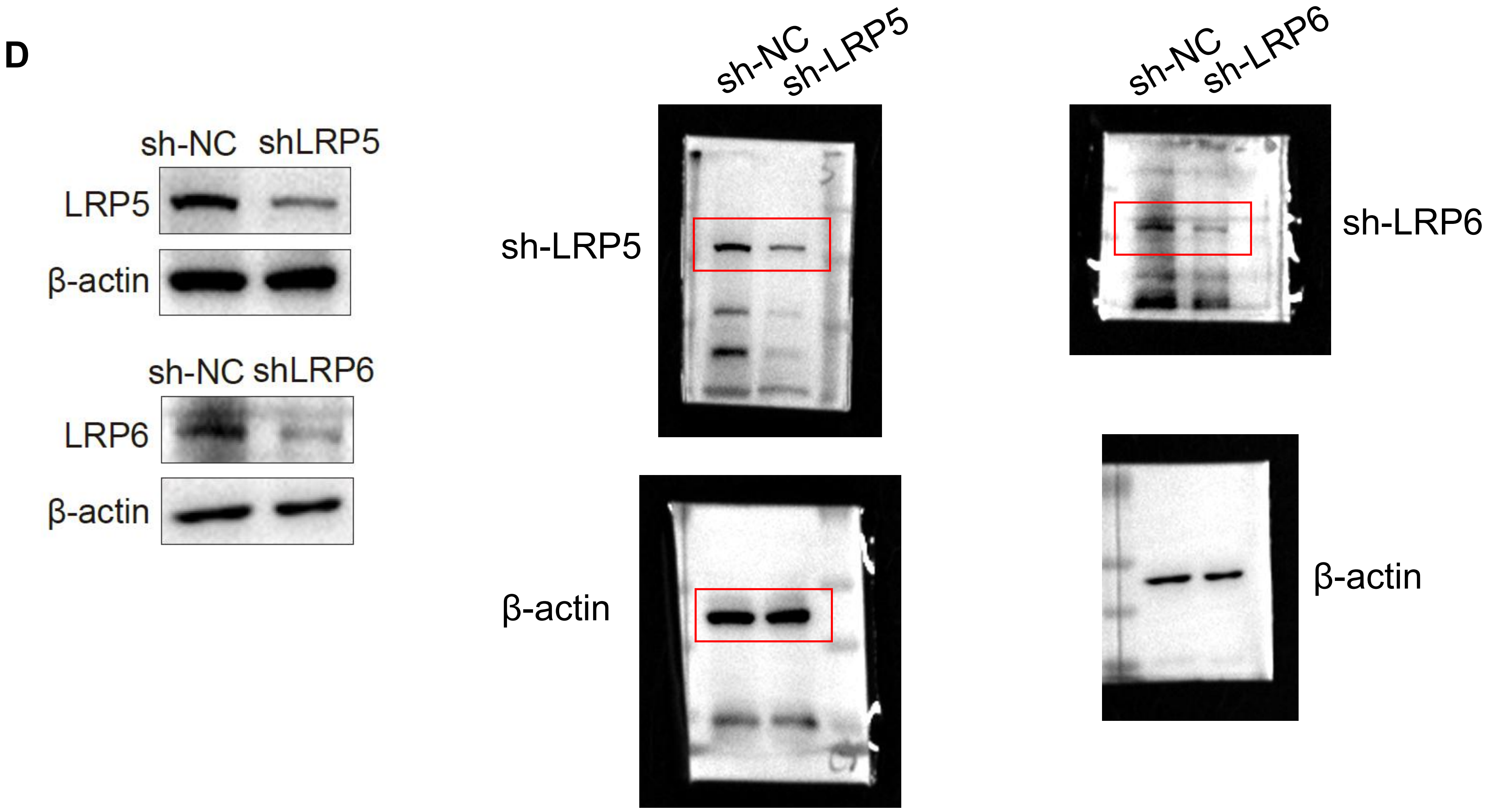

Supplement: Unedited blot and gel images [file jci-136-196722-s092.pdf]
